# Critical factors in achieving fine‐scale functional MRI: Removing sources of inadvertent spatial smoothing

**DOI:** 10.1002/hbm.25867

**Published:** 2022-04-13

**Authors:** Jianbao Wang, Shahin Nasr, Anna Wang Roe, Jonathan R. Polimeni

**Affiliations:** ^1^ Department of Neurosurgery of the Second Affiliated Hospital, Interdisciplinary Institute of Neuroscience and Technology, School of Medicine Zhejiang University Hangzhou China; ^2^ Athinoula A. Martinos Center for Biomedical Imaging Massachusetts General Hospital Charlestown Massachusetts USA; ^3^ Department of Radiology Harvard Medical School Charlestown Massachusetts USA; ^4^ Key Laboratory for Biomedical Engineering of Ministry of Education Zhejiang University Hangzhou China; ^5^ Division of Health Sciences and Technology Massachusetts Institute of Technology Cambridge Massachusetts USA

**Keywords:** cortical surface‐based analysis, fMRI preprocessing, high‐resolution fMRI, spatial blur, spatial specificity, ultra‐high field MRI, V2 stripes

## Abstract

Ultra‐high Field (≥7T) functional magnetic resonance imaging (UHF‐fMRI) provides opportunities to resolve fine‐scale features of functional architecture such as cerebral cortical columns and layers, *in vivo*. While the nominal resolution of modern fMRI acquisitions may appear to be sufficient to resolve these features, several common data preprocessing steps can introduce unwanted spatial blurring, especially those that require interpolation of the data. These resolution losses can impede the detection of the fine‐scale features of interest. To examine quantitatively and systematically the sources of spatial resolution losses occurring during preprocessing, we used synthetic fMRI data and real fMRI data from the human visual cortex—the spatially interdigitated human V2 “thin” and “thick” stripes. The pattern of these cortical columns lies along the cortical surface and thus can be best appreciated using surface‐based fMRI analysis. We used this as a testbed for evaluating strategies that can reduce spatial blurring of fMRI data. Our results show that resolution losses can be mitigated at multiple points in preprocessing pathway. We show that unwanted blur is introduced at each step of volume transformation and surface projection, and can be ameliorated by replacing multi‐step transformations with equivalent single‐step transformations. Surprisingly, the simple approaches of volume upsampling and of cortical mesh refinement also helped to reduce resolution losses caused by interpolation. Volume upsampling also serves to improve motion estimation accuracy, which helps to reduce blur. Moreover, we demonstrate that the level of spatial blurring is nonuniform over the brain—knowledge which is critical for interpreting data in high‐resolution fMRI studies. Importantly, our study provides recommendations for reducing unwanted blurring during preprocessing as well as methods that enable quantitative comparisons between preprocessing strategies. These findings highlight several underappreciated sources of a spatial blur. Individually, the factors that contribute to spatial blur may appear to be minor, but in combination, the cumulative effects can hinder the interpretation of fine‐scale fMRI and the detectability of these fine‐scale features of functional architecture.

## Graphical abstract

In order to evaluate the strategies to reduce spatial resolution losses during fMRI data preprocessing for high resolution surface‐based columnar mapping, we quantified inadvertent blur using both synthetic fMRI data and real fMRI data from human visual cortex, the spatially interdigitated V2 “thin” and “thick” stripes. Results surprisingly show that the simple method of volume upsampling can effectively preserve spatial resolution, and we also highlight less well‐known sources of spatial nonuniform blur that are present during acquisition. These findings uncover several underappreciated sources of the spatial blur, which can hinder the interpretation of submillimeter fMRI and the detectability of these fine‐scale features of functional architecture.

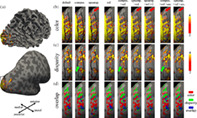

**Abbreviations**
EPI: echo‐planar imaging
fMRI: functional magnetic resonance imaging
FWHM: full width at half maximum
HCP: Human Connectome Project
MRI: magnetic resonance imaging
RMSE: root‐mean‐square error
TSTD: temporal standard deviation
UHF‐MRI: ultra‐high field magnetic resonance imaging
V1: primary visual cortex
V2: second visual cortex

## 1 INTRODUCTION

Ultra‐high field (≥7T) functional magnetic resonance imaging (UHF‐fMRI) has enabled the study of the fine‐scale functional architecture of the human brain *in vivo*, at the spatial scale of cerebral cortical columns (Cheng et al., 2001; Nasr et al., 2016; Yacoub et al., 2008) and layers (Huber et al., 2017; Muckli et al., 2015; Norris & Polimeni, 2019). Because the contrast‐to‐noise ratio of fMRI increases supralinear with field strength (Triantafyllou et al., 2005; Turner et al., 1993; Yacoub et al., 2001), this sensitivity boost provided by UHF‐fMRI can be used to enable imaging resolutions at submillimeter and/or subsecond scales. Although the ultimate “biological resolution” of the hemodynamic response to neural activity is still unknown, it appears that the hemodynamic response in the human brain can be used to localize the activity of these mesoscale features of neuronal organization. Indeed, features such as cortical columns have long been observed using intrinsic‐signal optical imaging techniques, which are also based on tracking hemodynamic changes that accompany neural activity (Grinvald et al., 1986; Lu & Roe, 2008; Roe & Ts'o, 1995; Valverde Salzmann et al., 2012). These measurements from intrinsic‐signal optical imaging have been shown to be well‐aligned with what can be observed with high‐resolution fMRI (Chen et al., 2007).

The main challenges today to resolve activation of distinct cortical columns and layers with fMRI are to sensitize the fMRI measurement to the hemodynamics of the microvasculature—which exhibits the closest relationship to neural activity—and to achieve sufficient imaging resolution. These targeted features of the functional architecture are, however, just at the edge of the imaging resolution that can be achieved with modern fMRI technologies (Polimeni & Wald, 2018). Therefore, any inadvertent smoothing that is imposed during data acquisition and/or preprocessing can lead to a failure to detect these features.

While the *nominal* resolution of acquisitions may appear to be sufficient to resolve these features, there are many well‐known sources of unwanted spatial blurring, such as T_2_* decay in EPI (Chaimow & Shmuel, 2016; Farzaneh et al., 1990) and partial Fourier acquisition (Zaretskaya & Polimeni, 2016), that result in losses of *effective* imaging resolution, which impede our ability to detect these features. What is less well appreciated is that data processing will also cause potentially avoidable losses in effective resolution (Glasser et al., 2013; Polimeni et al., 2018).

Spatial blurring induced by interpolation during preprocessing is the major contributor to losses in the effective resolution after the data have been acquired. For example, studies have reported spatial resolution losses caused by interpolation during geometric distortion correction (Renvall et al., 2016) and by motion correction (Grootoonk et al., 2000; Polimeni et al., 2018; Power et al., 2017). It is crucial to minimize these losses not only because they induce bias and artifacts such as regionally varying spatial blurring but they also decrease the reliability and reproducibility of the results. A recent study by Botvinik‐Nezer et al. (2020) compared the consistency of fMRI results generated by 70 independent experienced teams analyzing the same dataset and showed sizeable variation in their reported results; a post hoc investigation of the discrepant results suggested that the largest factor contributing to the inconsistency was implicit or unwanted spatial smoothing induced by data preprocessing.

Efforts have previously been made to address and minimize the effective resolution losses occurring during preprocessing. For example, in the Human Connectome Project (HCP), minimization of spatial blurring was achieved by minimizing spatial interpolation by composing all transformations applied to the data into one, thereby interpolating the data only once (Glasser et al., 2013, 2016). Another approach to minimize blurring is to upsample the image volume to a finer voxel grid to preserve spatial detail during interpolation—and, potentially, exploit the small head displacement (Allen et al., 2022; Kang et al., 2007; Zhang et al., 2015). Other similar approaches have focused on minimizing interpolation‐induced resolution losses, such as the use of higher‐order interpolants or nonlinear interpolation schemes.

Another important facet that has received less attention, which is particularly relevant for mapping cortical columns, is the potential resolution losses associated with projecting the volumetric fMRI data onto cortical surface meshes, which itself is a form of data interpolation that can lead to regionally varying losses in resolution (Kay et al., 2019; Polimeni et al., 2018).

Although strategies listed above have been proposed in various studies, their impact on the final resolution is not often quantified, and their ability to retain resolution at the fine spatial resolution needed to detect columnar organization has not been evaluated. The performance of these strategies may depend on the nominal imaging resolution and could be influenced by other aspects of the acquisition of these data—such as geometric distortions specific to UHF‐fMRI, the use of partial brain coverage for acquisition, and so on—and therefore should be evaluated for high‐resolution UHF‐fMRI data to better understand the limits of these methods in practice for this application.

Motivated by this, here our goal is to both evaluate and minimize several key sources of blurring imposed both during the data acquisition and analysis stages. These include aspects that have received less attention in past studies such as resolution losses imparted by projecting the volumetric fMRI data onto cortical surface meshes and the losses (and potential gains) in resolution associated with geometric distortion. We propose several practical methods to preserve spatial resolution during analysis and suggest a simple method for quantifying spatial resolution that can be applied to any preprocessing stream. Quantification methods such as this enable principled comparisons between preprocessing strategies and can provide valuable spatial “error bars” to use when evaluating results.

Both synthetic data and real data are employed in this study. We also consider the effects of image resolution on the accuracy of motion estimation, and demonstrate that the simple approach of upsampling the imaging data can be an effective way to limit resolution losses. To evaluate these approaches applied to our targeted application, imaging cortical columnar patterns, we exploit a favorable feature found in the columnar patterns within human extrastriate cortex: the fine‐scale spatial interdigitation of color selectivity “thin” and disparity selectivity “thick” stripes in human second visual (V2) cortex. This interdigitation, known from classic histology studies (Tootell et al., 1983), provides us with valuable “ground truth”; that is, the two independently localized stripe (column) types should exhibit minimal spatial overlap, which allows us to meaningfully evaluate the effects of spatial blur in these data across different preprocessing strategies. Because these high‐resolution data are typically acquired across experimental sessions, and these two V2 columnar sub‐systems were imaged independently in different sessions, our data also allow us to evaluate the accuracy of aligning multi‐session data.

A preliminary account of this study was previously presented in abstract form (Wang et al. 2021).

## 2 METHODS

### 2.1 Participants

Three human subjects (two females; age range, 24–28 years) participated in this study. Written informed consent was obtained from each participant before the experiment, and all experimental procedures were performed in accordance with our institutionally approved Human Research protocol and federal guidelines. Data from these three subjects have been previously published; see our previous study (Nasr et al., 2016) for additional details.

### 2.2 Visual stimuli

Visual stimuli were presented via an LCD projector (1024 × 768‐pixel resolution, 60 Hz refresh rate) onto a rear‐projection screen, viewed through a mirror mounted on the receive coil array. MATLAB 2013a (MathWorks) and the Psychophysics Toolbox (Brainard, 1997; Pelli, 1997) were used to present visual stimuli. In order to map V2 “thin” and “thick” stripes, an experiment targeting color selectivity and an experiment targeting disparity selectivity were performed in separate experimental sessions; both experiments were conducted over two sessions, thus four experimental sessions were conducted in total for each participant.

For the color experiment, sinusoidal drift gratings were presented either in iso‐luminance chromatic or achromatic luminance with low spatial frequency (0.2 cycle/°) and temporal frequency (4°/s). For the disparity experiment, random‐dot stereograms (RDSs) based on red or green dots (0.09° × 0.09°) were presented against a black background, which were perceived stereoscopically as an array of cuboids that sinusoidally varied in time in depth (±0.22°) or at a fixed depth in a frontoparallel plane. Each experimental session consisted of 8–12 BOLD fMRI runs (see below) to improve detection sensitivity in each individual subject. Details of stimulus presentation, fixation task, and luminance calibration can be found in our previous study (Nasr et al., 2016).

### 2.3 Data acquisition

#### 2.3.1 Functional data

Functional data were acquired using a 7T whole‐body scanner (Magnetom, Siemens Heathineers) equipped with body gradients (maximum gradient strength, 70 mT/m; maximum slew rate, 200 T/m/s), with a custom‐built 32‐channel helmet receive coil array and a birdcage volume transmit coil (Keil et al., 2010). BOLD fMRI data were acquired using a 2D single‐shot gradient‐echo EPI protocol at 1‐mm isotropic nominal resolution using the following protocol parameter values: TR = 3000 ms, TE = 28 ms, matrix size = 192 × 192, bandwidth = 1184 Hz/pix, nominal echo spacing = 1 ms, partial Fourier = 7/8, flip angle = 78°, slice number = 44, acceleration factor = 4 with GRAPPA reconstruction and FLEET‐ACS data (Polimeni et al., 2016) with 10° flip angle. Prior to fMRI data acquisition, RF transmit voltage amplitude was calibrated to the visual cortex region of interest.

#### 2.3.2 Retinotopic mapping

In order to functionally define the cortical area boundaries within the visual cortex, including the borders of V2 (needed for our analysis of columnar sub‐system or stripe overlap), standard retinotopic mapping was performed for each subject. These retinotopic mapping data were acquired in each participant using a 3T Siemens scanner (Tim Trio) and the vendor‐supplied 32‐channel receive coil array, using single‐shot gradient‐echo BOLD‐weighted EPI protocol with nominally 3.0 mm isotropic voxels and the following protocol parameter values: TR, 2000 ms; TE, 30 ms; flip angle, 90°; matrix, 64 × 64; BW, 2298 Hz/pix; echo‐spacing, 0.5 ms; no partial Fourier; FOV, 192 × 192 mm; 33 axial slices covering the entire brain.

#### 2.3.3 Anatomical data

Structural (anatomical) data were also acquired using a 3T Siemens scanner (Tim Trio) using a 3D T_1_‐weighted MPRAGE sequence with protocol parameter values: TR = 2530 ms, TE = 3.39 ms, TI = 1100 ms, flip angle = 7°, bandwidth = 200 Hz/pix, echo spacing = 8.2 ms, voxel size = 1.0 × 1.0 × 1.33 mm, FOV = 256 × 256 × 170 mm. These anatomical data served as an anatomical reference for all fMRI data.

### 2.4 Data preprocessing and analysis steps common to all evaluations

#### 2.4.1 Standard preprocessing

##### Anatomical reference data preprocessing

In order to visualize the columnar data on the unfolded cerebral cortical gray matter (GM), surface reconstructions were automatically generated using FreeSurfer (version 6 (http://surfer.nmr.mgh.harvard.edu/; Fischl, 2012). To evaluate the effects of intra‐cortical smoothing (see next section), we also automatically generated, in addition to the standard pial surface (i.e., the gray matter border with the surrounding cerebrospinal fluid or CSF) and the white matter (WM) surface reconstructions, a family of 11 intermediated equidistant surfaces spaced at intervals of 10% of the cortical thickness, which consisted the WM‐GM (surface 0) interface surface, the GM‐CSF (surface 10) interface surface, and 9 intermediate surfaces within gray matter.

##### Functional data preprocessing

The main fMRI data preprocessing includes the following. (1) fMRI data motion correction, including motion estimation. Each fMRI run was independently aligned to the middle frame of the run (the reference frame) using rigid‐body alignment. (2) fMRI data registration to the anatomical reference data. A rigid‐body transformation was computed again from the middle frame of each run (the reference frame) to the T_1_‐weighted anatomical image volume using boundary‐based registration (BBR; Greve & Fischl, 2009). The accuracy of this registration between the fMRI volume to the anatomical volume was verified visually. Note that no nonlinear warping or distortion correction was applied due to the low level of distortion in these accelerated EPI data seen within the calcarine sulcus (see Section 4). (3) Projection of the fMRI volumetric data onto the cortical surface mesh derived from the anatomical reference. The motion‐corrected fMRI data were projected to the WM‐GM surface by applying the transformation computed during anatomical registration. No explicit spatial smoothing in volume space was applied. Details of the interpolation used for the motion correction and for the surface projection, both of which are a focus of the current study, are provided further below.

#### 2.4.2 Standard statistical analysis

To detect functional activation in the fMRI data, a standard statistical analysis, based on a general linear model (GLM) for the expected changes in BOLD signal with activation, was applied to the preprocessed time series data. A canonical hemodynamic response was assumed (i.e., a gamma function with 2.25 s hemodynamic delay and 1.25 s dispersion). The estimated activation maps from across runs or across sessions, where applicable, were always combined using a fixed‐effect analysis.

#### 2.4.3 fMRI data visualization

To visualize the activation patterns, including activations tucked within buried sulci of the extrastriate cortex, activation maps were displayed on an “inflated” surface representation, with the sulcal and gyral patterns indicated in grayscale.

### 2.5 Preprocessing steps evaluated for preserving accuracy

Our goals in this study are to evaluate methods for both preserving spatial accuracy and quantifying the resulting resolution losses. We, therefore, compared results generated several times via different strategies. Here we consider resolution losses attributed to preprocessing and those due to the acquisition process. Strategies evaluated to reduce unwanted resolution loss include reducing voxel grid spacing through upsampling, various higher‐order and nonlinear interpolation algorithms, one‐step resampling, and surface mesh refinement. The effects of imaging resolution and voxel grid spacing on motion correction accuracy and the spatially varying blur caused by geometric distortion during acquisition were also characterized.

#### 2.5.1 Upsampling of fMRI volumes

Several fMRI preprocessing steps involve the spatial transformation of the fMRI volumes. This includes motion correction and also steps such as geometric distortion correction, which result in either rigid or nonrigid transformations. For these transformations, voxel data are in general shifted off of the voxel grid, which necessitates resampling and therefore some form of interpolation, which results in spatial blurring and spatial resolution losses. Because the spatial extent of the interpolation kernel is a function of the voxel grid spacing (equivalently, the voxel size), one method to reduce the spatial blur associated with interpolation is to simply upsample the fMRI volume prior to resampling.

Additionally, upsampling the fMRI volumes could also potentially improve the estimation of motion parameters in time‐series data. This is mainly because a finer grid spacing can provide several motion estimation algorithms with a larger number of voxels that contribute to the estimation as well as more potential translations and rotations of the volumetric data to evaluate when computing the spatial similarity of a given displaced frame of data with the reference frame.

To test the effects of fMRI volume upsampling, we upsampled the native (nominally) 1‐mm isotropic resolution data by a factor of 2 in the linear dimension to a 0.5‐mm isotropic grid, resulting in a voxel size that is eight times smaller in volume (and thus eight times more voxels per volume) prior to motion correction. The fMRI data were interpolated onto this finer voxel grid using several interpolation algorithms: nearest‐neighbor, cubic, and linear interpolation. To isolate the effects of upsampling on resolution losses from interpolation, we computed all transformations of the fMRI data on the original 1‐mm isotropic volumes (including motion correction and anatomical registration) and applied these transforms to the upsampled data. We then compared resolution losses seen in the original 1‐mm isotropic data and on the data resampled to the 0.5‐isotropic grid with each of the three volume‐upsampling interpolation methods.

#### 2.5.2 Transformation composition

Best‐practice fMRI preprocessing seeks to reduce resolution losses caused by interpolation, and the recommendation is to minimize the number of times the fMRI data are interpolated (Glasser et al., 2013). The amount of implicit spatial blurring imparted by multiple sequential steps of interpolation can be minimized by mathematically composing the transformations such as the transformation of each fMRI frame to the fMRI reference frame used in motion correction with the transformation of the fMRI reference frame to the anatomical reference. In order to evaluate the impact of transformation composition on fMRI resolution, we implemented a transformation composition that transformed each fMRI frame into the anatomical reference, which allowed us to project each frame of fMRI data directly onto the cortical surface mesh. To achieve this, first, the spatial transformations corresponding to the motion estimation were generated from the motion parameters. Second, the transformations of the motion correction and anatomical registration were composed. Finally, the composed transformation was used to project each fMRI voxel onto the cortical surface mesh. In this approach, the fMRI data undergo only a single interpolation (i.e., when the data are projected onto the cortical surface mesh) and no volumetric interpolation is applied to the fMRI data.

For comparison, we computed both the activation map computed using the standard approach of sequentially applying the two transformations (resulting in two interpolation steps) with the activation map computed using the composed transformation.

#### 2.5.3 Refinement of surface mesh

When projecting volumetric data onto a surface mesh, it is important for the spacing of the mesh vertices to be sufficiently small that it can accurately represent the volumetric data. In general, this “resolution” or vertex spacing of the surface mesh is a function of the resolution of the anatomical imaging data used to reconstruct the surface mesh, so smaller anatomical voxels will lead to finer meshes (Zaretskaya et al., 2018). Today, high‐resolution fMRI voxels are similar in size or smaller than typical MPRAGE voxels, and so it is possible that the surface mesh spacing is insufficiently small for these fMRI data. If the mesh vertex spacing is larger than the fMRI voxel size, it is possible that fMRI voxels sampling the cerebral cortex are not represented on the surface mesh, resulting in lost data or “holes” in the activation map represented on the cortical surface. This can also result in small‐scale geometric distortions of the fMRI data projected onto the surface. These two issues can be minimized by refining the surface mesh so that the distances between neighboring vertices are smaller than the fMRI voxel grid spacing.

In order to evaluate the effect of surface mesh refinement on the surface‐based representation of the fMRI data, we iteratively refined the native surface mesh, with each iteration adding new vertices, thereby increasing vertex density and decreasing vertex spacing. For our refinement algorithm, we employed a simple form of the surface subdivision known as the “butterfly” method under which each refinement iteration consisted of inserting a new vertex at the mid‐point of each mesh edge, then connecting the three new vertices in each mesh triangle, which subdivided each original triangle into four new, smaller triangles. Thus, because FreeSurfer generates surfaces for which the number of faces is roughly twice the number of vertices, each iteration also increased the number of vertices by approximately a factor of 4 and decreased the vertex spacing by about a factor of 2, without changing the geometry of the surface or the positions of any of the original vertices. Here we performed four successive iterations of mesh refinement. Then the fMRI volumetric data were projected onto the refined surface meshes and interpolated with trilinear interpolation. We also calculated the number of unique fMRI voxels projected onto each surface mesh to quantify the number of recovered voxels with each step of refinement (Section 2.6.2).

#### 2.5.4 Intracortical smoothing

Although spatial smoothing can help increase signal‐to‐noise ratio (SNR) and boost sensitivity, typical volumetric 3D smoothing kernels applied to the cortical gray matter can increase partial volume effects, leading to noise contamination from surrounding CSF, signal dilution from WM, and a mixing of activation from pial veins and the parenchyma, which again can lead to displacement of activation and a general loss of spatial specificity.

In order to increase temporal signal‐to‐noise ratio (tSNR) in fMRI data while minimizing loss of spatial specificity, anatomically informed spatial smoothing can be applied that restricts the smoothing to be within the cortical gray matter ribbon using the surface mesh‐navigated spatial smoothing method (Blazejewska et al., 2019). This method allows for smoothing relative to the natural coordinate system of the cortex, either parallel to the cortex, perpendicular to the cortex, or in the form of a “steerable” 3D kernel that follows curvature of the folding pattern.

Because the goal of the present study to is map columnar organization, here we evaluate anatomically informed smoothing in the direction perpendicular to the cortex to smooth within the columns. This approach leverages strong prior information that similar fMRI responses to our stimuli are expected to be more similar in the direction perpendicular to the cortex than they are in the direction parallel to the cortex. Here intracortical smoothing is performed only in the radial direction (i.e., with no smoothing in the tangential direction) with an extent that is limited to the bottom 30% of the cortical ribbon, that is, from the 0% surface at the white‐matter interface to the surface at 20% of the relative cortical thickness (as defined in Section 2.4), which allows us to retain spatial resolution for mapping the columnar patterns while increasing SNR through exploiting the prior knowledge about the columnar organization. This conservative radial smoothing kernel was chosen to retain the spatial specificity, because of the known losses of spatial specificity for at the pial surface due to large vein contamination in gradient‐echo BOLD (Nasr et al., 2016; Polimeni et al., 2010). Considering both our voxel size relative to the thickness of the visual cortex, conservatively smoothing voxels within the bottom 30% of the cortical thickness would capture one to two voxels, which would be expected to capture the deep and middle cortical layers and, in some locations along the folded cortex, may reach the superficial layers but would rarely reach the pial surface. We compared the results of our columnar mapping with and without this intracortical smoothing applied to the fMRI data.

### 2.6 Evaluation of effects of preprocessing on spatially varying smoothing

To evaluate the effects of the various preprocessing strategies summarized above on spatial blurring, we utilized both synthetic data and real fMRI data as follows.

#### 2.6.1 Quantification of spatial blurring using synthetic white noise

To quantify the level of spatial blurring induced during preprocessing, and the spatially varying pattern of this blur, we followed a procedure employed previously based on generating a synthetic i.i.d. white‐noise time series matching the resolution of the functional data (prolonged in time dimension to reduce variance of white‐noise across voxels), and subjecting this 4D noise volume to various preprocessing steps (Polimeni et al., 2018; Renvall et al., 2016). This method is akin to a Monte Carlo simulation, which is often applied to characterize how various analysis steps introduce spatial correlation through blurring (Hagler et al., 2006). The amount of spatial blur can be determined numerically by the mapping between the resulting temporal standard deviation (TSTD) and the explicitly applied 3D Gaussian smoothing kernels with varying smoothing capacity ranging from 0.1 to 3.9 FWHM. Here we derived this mapping between TSTD and FWHM by simply applying a 3D Gaussian smoothing function with various widths (using the fslmaths command of the FSL toolbox) and recorded for each FWHM the resulting TSTD of the smoothed synthetic noise data. This provided a look‐up table that maps the measured TSTD of the processed noise data to the equivalent FWHM of the induced 3D smoothing.

##### Quantification of spatial blurring induced by motion correction for different interpolation algorithms, with and without volume upsampling

To compare the extent of spatial blurring introduced during motion correction, both for our original 1‐mm fMRI data and the upsampled 0.5‐mm fMRI data, we synthesized white noise data on a 1‐mm grid, then upsampled these data to a 0.5‐mm grid. For this experiment, interpolation was performed for two purposes: during upsampling, and during motion correction. We evaluated the induced blurring for three different interpolation algorithms used for upsampling (plus the case of no upsampling) and for four different interpolation algorithms used for motion correction, resulting in a total of 16 tests. For each test, the FWHM was calculated by finding the correspondence to the calculated TSTD using the look‐up table described above and averaged across all voxels within the volume.

##### Quantification of spatial blurring induced by projecting volumetric fMRI data onto cortical surface meshes and the effects of composed transformations

Because many aspects of functional architecture of the cerebral cortex are naturally organized into 2D columnar or topographic maps embedded in the cortical surface, for many studies, it is necessary to project the fMRI data onto the cortical surface representation both for visualizing and analyzing these 2D functional maps. To represent the volumetric fMRI data on the cortical surface, the so‐called volume‐to‐surface projection assigns fMRI voxels to surface mesh vertices, and then once the data are represented on vertices they can be visualized or rendered using various approaches. Because the surface mesh is typically an irregular polyhedral mesh (e.g., a triangulation with nonuniform vertex spacing and varying triangle areas) the closest mesh vertex to a particular voxel centroid is in general offset by a small amount, and thus the projection also requires an interpolation of the volumetric data—in this case, instead of interpolating data from one voxel grid onto another voxel grid, the data are interpolated from a voxel grid onto an irregular mesh. Note that the closest mesh vertex is determined using the transformation computed during the functional‐to‐anatomical registration.

There are several methods for this interpolation, and different interpolation strategies are expected to induce different amounts of spatial blurring. For example, in trilinear interpolation each vertex value is calculated as a weighted sum of nearby voxel values, which helps to infer what the fMRI intensity is at a vertex situated “in between” two or more voxels. However, this will introduce spatial blurring and correlations into the functional data represented on the surface. In nearest‐neighbor interpolation, each voxel is projected to all of the vertices it intersects, which leads to some spatial distortion of the functional data depending on the arrangement of the vertices. However, it avoids spatial blurring and does not introduce correlations that are not present in the original volumetric data. Although these surface interpolation effects are known, the effects are less quantified compared to volume interpolation effects, especially for high spatial resolution fMRI data.

To quantify the surface projection‐induced blurring of the fMRI data, we took the following steps: (i) we projected 4D synthetic white noise data onto the white matter surface mesh using both nearest‐neighbor and trilinear interpolation methods (using the FreeSurfer command mri_vol2surf). (ii) Each vertex was assigned a time‐series of noise intensities, then the temporal standard deviation was calculated from these projected noise data for each vertex. (iii) To determine the effective 3D smoothing kernel corresponding to these TSTD values, we used the same lookup table described above. While these resolution losses are induced by projection onto the 2D surface mesh, and so they can be characterized by the effective 2D smoothing kernel, which could be readily calculated using the procedure described above (using a 2D surface‐based smoothing approach), here to keep this evaluation comparable to the evaluation of volumetric smoothing we chose to quantify the blurring in terms of the effective 3D smoothing; naturally, there is a simple relationship between the effective isotropic 3D smoothing extent and the effective isotropic 2D smoothing extent.

Because the fMRI data typically undergo several volume transformations followed by a surface projection during preprocessing, we quantified the effect of composing the volume transformations and surface projection so that the data would be interpolated only once (i.e., when projecting onto the surface). In this evaluation, again we used 4D white noise data and tested the effective blurring induced by the conventional approach in which volumetric transformation (in this case, motion correction) and interpolation is followed by surface projection and interpolation, and the proposed approach in which the volumetric transformation was composed with surface projection such that each frame of uncorrected fMRI data were projected directly onto the cortical surface mesh.

#### 2.6.2 Quantification of precision/accuracy trade‐off on volume to surface projection

Although nearest‐neighbor interpolation, compared to trilinear interpolation, helps reduce smoothing when projecting volumetric data onto surface meshes, it can cause small‐scale distortions and missing voxels in the data if the mesh is too coarse—which means that nearest‐neighbor interpolation induces potentially less variance but more bias, thus choosing an interpolation method requires balancing a bias‐variance trade‐off. These small‐scale distortions can be minimized by refining the surface mesh so that the distances between neighboring vertices are smaller than the voxel grid spacing. Here, because the activation map is a prediction of the location of neuronal response after preprocessing, prediction errors may arise. There are two kinds of prediction errors related to losses of accuracy and losses of precision—that is, related to either bias or variance. In our case, bias is related to displacements and distortions in the estimated stripe pattern, and variance is related to noise and to sensitivity to detect activation. These terms are discussed in more detail in Section 4.11.

To quantify the proportion of voxels lost during the volume‐to‐surface projection, a synthetic volume in which the value of each voxel was assigned a unique voxel index was generated, and this volume was then projected onto the surface mesh (using the same computed registration) using nearest‐neighbor interpolation. This was performed for the original surface mesh and for refined surfaces generated with a refinement factor ranging from 1 to 4 (as described above in Section 2.5.3). Then each unique voxel projected to the surface was identified, and the proportion of unique voxels was calculated for the original and refined surfaces. While the proportion of unique voxels will of course depend on the voxel grid spacing, here we aim to demonstrate a method to evaluate how refinement can help to retain more of the acquired fMRI voxels when projecting onto cortical surface meshes.

### 2.7 Other factors influencing spatial accuracy

#### 2.7.1 Effect of imaging resolution on motion estimation accuracy

Because imperfect motion estimation will also result in loss of spatial accuracy when performing motion correction, we also quantified the influence of imaging resolution on motion parameter estimation accuracy. This was achieved by (1) synthesizing a 4D volume with known motion by applying measured rigid‐body motion parameters (generated from the motion estimation of our acquired 1‐mm fMRI data) to a single 3D frame of our 1‐mm fMRI data, and then (2) resampling the resulting synthetic 4D volume with known motion to generate 4D volumes with larger voxel sizes (2 and 3 mm) and with a smaller voxel size (0.5 mm) and known motion. This first test evaluates the effect that resampling (upsampling or downsampling) data acquired natively at 1‐mm resolution has on motion estimation accuracy.

Subsequently, to illustrate whether motion estimation accuracy could potentially be improved by acquiring even higher resolution fMRI data, again rigid‐body motion parameters from acquired fMRI data were applied to generate synthetic data with known motion, but in this case, the motion was applied to a single 3D frame of our natively 1‐mm fMRI data after resampling to a 3D volume with larger voxel sizes (2 and 3 mm) and with a smaller voxel size (0.5 mm). In this case, the resampling was performed prior to applying the known motion to generate 4D volumes with known motion. This second test evaluates the effect that resolution of the acquired data has on motion estimation accuracy.

For both tests, motion was then estimated from the synthetic 4D volumes with known motion, and the accuracy of these estimates was determined by the differences between the estimated motion parameters and known motion parameters, and the error was summarized as the root‐mean‐square error (RMSE).

#### 2.7.2 Quantification of spatial resolution losses caused by geometric distortion

Not only can the resolution of the fMRI data vary spatially due to different preprocessing strategies, but it can also vary across brain regions due to geometric distortion. Geometric distortion, such as well‐known macroscopic susceptibility gradient induced distortion in EPI data, not only displaces fMRI voxels but also can expand or compress voxel sizes, leading to another form of spatially varying resolution across the fMRI volume. In order to illustrate this effect, we considered gradient nonlinearity distortion, which varies with the design of the gradient coil, as an example. To increase gradient coil performance, often the constraint that the gradient field used for image encoding is linear over space is relaxed, resulting in gradients that are a nonlinear function of space (Schmitt, 1985; Langlois et al., 1999). These gradient nonlinearities cause spatial localization errors that manifest as image distortion and voxel size variation, which are not accounted for in conventional image reconstruction that assumes a linear gradient field, and these geometric errors are a function of position of the head relative to isocenter.

The effects of these distortions have been illustrated recently (Yamamoto et al., 2021) but their influence on spatial resolution has not been quantified. Using the nonlinearity of several modern gradient coils provided by the scanner manufacturer, we applied the corresponding gradient nonlinearity warping to an example distortion‐free anatomical dataset, assuming that the head was positioned in the same location centered within each gradient coil. Then, we evaluated the voxel compression/expansion imparted by several modern gradient coil sets by computing the Jacobian determinant of the deformation induced by gradient nonlinearity.

### 2.8 Evaluation of spatial specificity through imaging nonoverlapping columnar subsystems

Besides using synthetic data to evaluate the effects of different preprocessing strategies on spatial blurring, we also quantified these effects in a way that is directly relevant to high‐resolution fMRI studies by investigating how the various strategies considered above influence our ability to detect spatially segregated and nonoverlapping fine‐scale patterns of functional architecture, the V2 “thin” and “thick” stripes. For this, we evaluated the preprocessing steps described in Section 2.5 to demonstrate the effect of each on the accuracy of detecting these stripes by measuring the overlap between the thin and thick stripes, which are known from histological studies to be nonoverlapping (Tootell et al., 1983).

To achieve this goal, the two columnar activation maps were computed following different preprocessing strategies, that is, the default preprocessing and preprocessing using multiple steps of interpolation with volume upsampling, one‐step interpolation from composed transformations as well as surface refinement, and intracortical smoothing. Then combinations of these strategies were also evaluated including (1) volume upsampling + surface refinement, (2) composed transformations + surface refinement, (3) volume upsampling + intracortical smoothing, (4) composed transformations + intracortical smoothing, (5) volume upsampling + surface refinement + intracortical smoothing, and (6) composed transformations + surface refining + intracortical smoothing. (The preprocessing strategies based on composed transformations and volume upsampling are were not combined because volume upsampling provides no benefits when performing single‐step interpolation onto the cortical surface mesh.)

Because strategies that result in less spatial blurring should produce “thin” and “thick” stripes with reduced overlap, we required a means to quantify overlap through quantifying the spatial borders of each columnar sub‐system. This could be achieved through thresholding the corresponding activation maps. To address the influence of the chosen threshold on the overlap quantification, binarized activation maps were generated using multiple thresholds (*p* =  0.05, 0.01, and 0.001), and activation clusters with a cluster size below two vertices were considered to be likely false‐positive activations due to noise and removed from the binarized activation maps. The Dice Coefficient (Dice, 1945) across the different threshold for different strategies was calculated to quantify overlap between the “thin” and “thick” stripe sub‐systems.

## 3 RESULTS

### 3.1 Effect of volume upsampling on spatial blur

**FIGURE 1:**
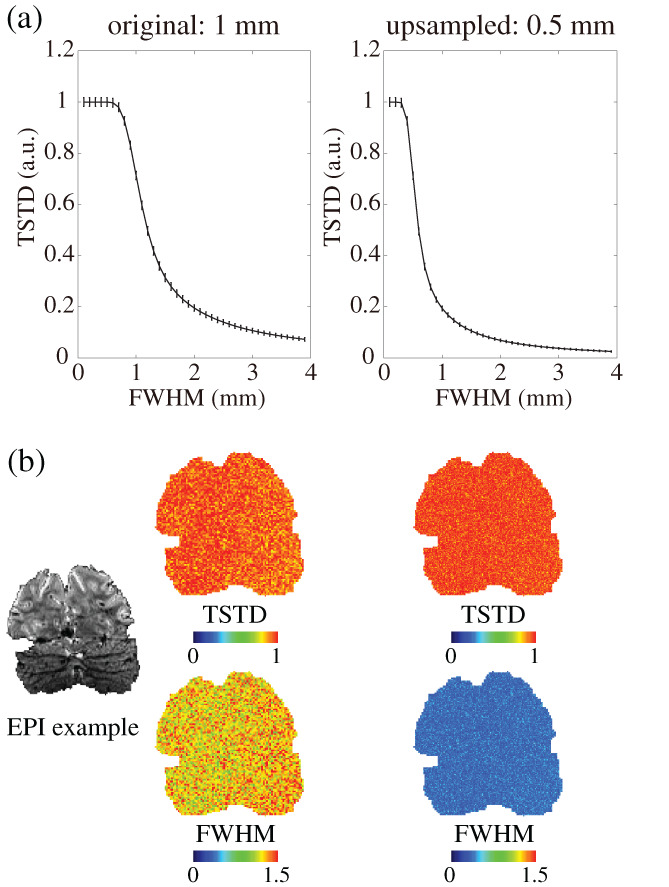
Evaluation of the effect of volume upsampling on effective spatial resolution. (a) Correspondence between TSTD and FWHM at original 1‐mm voxel grid and upsampled 0.5‐mm voxel grid. Error bars indicate variability across voxels which is influenced by the number of time point samples. (b) Left and right columns show the TSTD calculated from white noise after applying motion correction and the corresponding FWHM at the original 1‐mm and at the upsampled 0.5‐mm voxel grid. Cold color indicates lower value of TSTD/FWHM; warm color indicates higher value of TSTD/FWHM (inset shows the example EPI slice in coronal view)

In order to illustrate the correspondence between the applied 3D smoothing kernel (FWHM) and TSTD for synthetic white noise data, look‐up tables were calculated and the transfer function between TSTD and FWHM is shown in Figure 1a for a 1‐mm grid and for a 0.5‐mm grid. As depicted in the graph, the more spatial blur is applied to the image volume, the lower the noise TSTD, which indicates how TSTD can be used as an index to evaluate the amount of the spatial blur for each voxel. Error bars represent the variance of estimated TSTD across all of the voxels within the brain. This variance can be reduced by increasing the number of time points generated in the 4D synthetic white‐noise data, at the cost of increased computation time. The plateau at small values of TSTD corresponds to smooth kernels whose width is smaller than the voxel grid size, which indicates how the grid size chosen will influence the ability to capture fine‐scale spatial blur. This can be improved by moving to finer grid sizes, again at the cost of increased computation time. Comparing the transfer function computed for the 1‐mm grid and for the 0.5‐mm grid shown in Figure 1a, the extent of this plateau decreases, and the absolute amount of spatial smoothness becomes smaller, when using a finer grid size.

To demonstrate how upsampling the image volume can reduce spatial blur caused by interpolation during preprocessing, we evaluated blurring induced in the volume by applying motion correction to the synthetic white‐noise data for both the 1‐mm grid and for the 0.5‐mm grid, and present the TSTD and FWHM in an example coronal EPI slice to illustrate the mapping from TSTD to FWHM. These results show that, while the resulting TSTD values are similar between the two grids tested, when using the appropriate lookup table, we can see that the smoothing of the data in absolute units of mm is lower for the upsampled data on the 0.5‐mm grid  than it is in the 1‐mm data, as expected (Figure 1b).

### 3.2 Effects of interpolation algorithms used for volume upsampling and for spatial transformation

**FIGURE 2:**
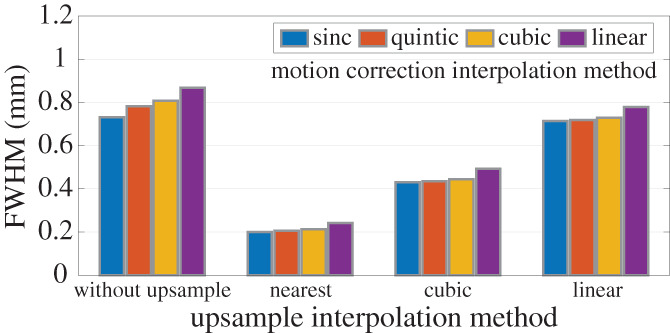
Effect of various interpolation methods used for volume upsampling and for spatial transformation. Each group of bars represents the smoothing induced by a specific interpolation algorithm (nearest‐neighbor, cubic, and linear interpolation) applied during volume upsampling prior to motion correction, and each color represents a specific interpolation algorithm (sinc, quintic, cubic, and linear interpolation) applied during motion correction

Although volume upsampling can reduce spatial blur induced when applying spatial transformations to the 3D image data, the act of upsampling itself requires an interpolation, and the choice of interpolation algorithm used for upsampling also has an effect on the total resolution loss—and if the interpolation algorithm used for upsampling is not chosen appropriately this interpolation step will counteract and potentially remove altogether any benefits of volume upsampling. To illustrate this interaction between the interpolation used for upsampling and the interpolation algorithm used for spatial transformation, we upsampled the synthetic volumes using various interpolation algorithms and then applied motion correction again using various interpolation algorithms. Note that, in general, different interpolation algorithms are available for volume upsampling and for spatial transformations. As can be seen in Figure 2, spatial blurring varies substantially across the different interpolation algorithms tested, and low‐order interpolation, trilinear for example, induces the most severe spatial blur, as expected. Importantly, applying linear interpolation during upsampling nearly cancels the benefits of upsampling for retaining resolution when applying the spatial transformation.

These data suggest that resolution losses due to interpolation happen not only when applying spatial transformations in volume space, but can also occur while projecting the volume data onto surface meshes, and surface refinement is in this case analogous to volume upsampling. This will be addressed in the next section.

### 3.3 Effects of composing transformations

**FIGURE 3:**
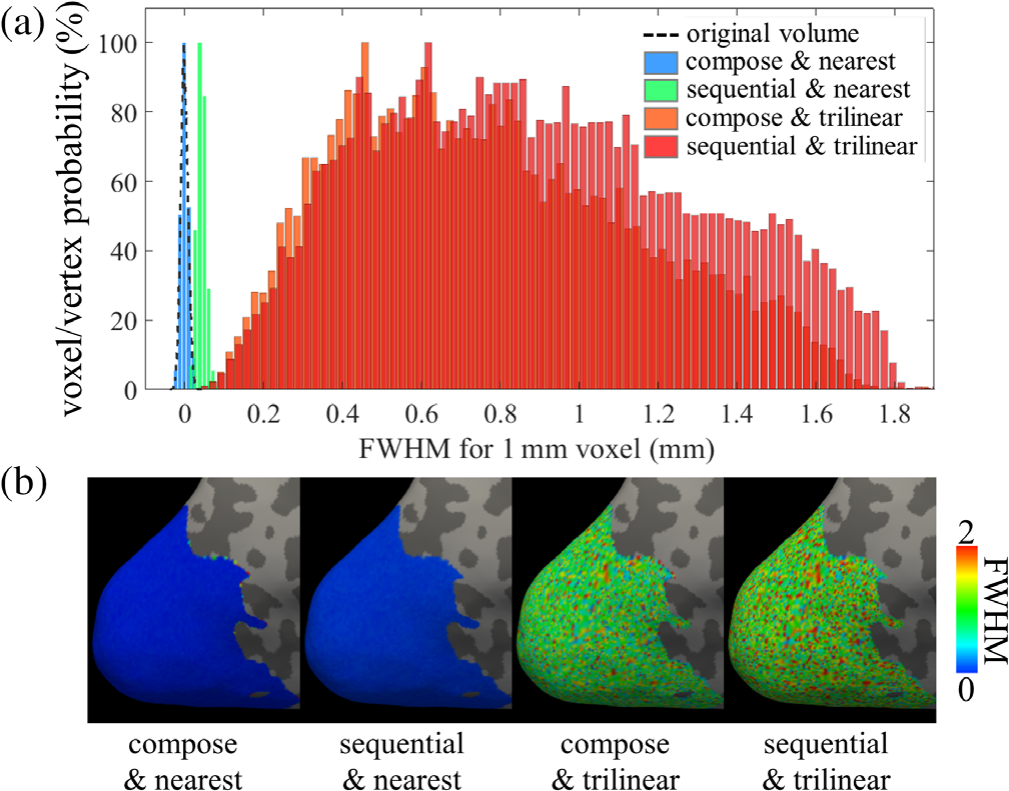
Total spatial blur induced in fMRI data projected onto the cortical surface, either using sequential transformations versus composed transformations, with either nearest‐neighbor or trilinear surface interpolation. (a) Histograms of the FWHM measured after all transformations are applied to the fMRI data projected onto the cortical surface, for different strategies of applying the transformation and projection. The dashed curve shows the original distribution of voxels without any interpolation, for reference. (b) The FWHM map projected onto the cortical surface is shown on visual cortex. Cold colors indicate low‐FWHM values and warm colors indicate high‐FWHM values

To evaluate to what extent spatial blur can be influenced by composing spatial transformations with surface projection, and the effects of surface interpolation algorithms, we projected the synthetic 4D white noise data onto the cortical surface mesh either by composing spatial transformations and projection or by applying spatial transformations and projection sequentially. Two different volume‐to‐surface interpolation algorithms were also compared. As expected, composing spatial transformations and projection indeed reduces the spatial blur as seen both in the histogram shown in Figure 3a and the spatial maps shown in Figure 3b. However, the effect of composed versus sequential transformation was small compared to the effect of interpolation. Compared to nearest‐neighbor interpolation, trilinear interpolation induces far higher spatial blur. Furthermore, and perhaps more importantly, trilinear interpolation induces a broader range of spatial smoothness as can be seen readily in the histograms of Figure 3a, leading to a higher variability across the cortex of effective spatial resolution. With nearest‐neighbor interpolation, the distributions of FWHM for composing transformation are less than 0.1 mm on average for this example data.

It should also be noted that, while nearest‐neighbor interpolation helps reduce smoothing when projecting onto surfaces, it can cause small‐scale spatial distortions in the data if the mesh is course, that is, there is a bias‐variance trade‐off that must be considered. This small‐scale distortion can be reduced by refining the surface mesh, as described in the next section.

### 3.4 Refinement of the surface mesh reduces the number of missed voxels

To test whether projecting fMRI data onto typical cortical surface meshes can result in lost fMRI voxels and thus missing data, and how this loss depends on both voxel grid and mesh vertex spacing, we counted the number of unique voxels projected onto the surface for various surface mesh resolutions. (Here, voxel index values, which are integers, were projected to the surface, and thus nearest‐neighbor interpolation was used to preserve their integer values.) The number of the lost voxels was then quantified by counting the number of unique values on the surface mesh.

**FIGURE 4:**
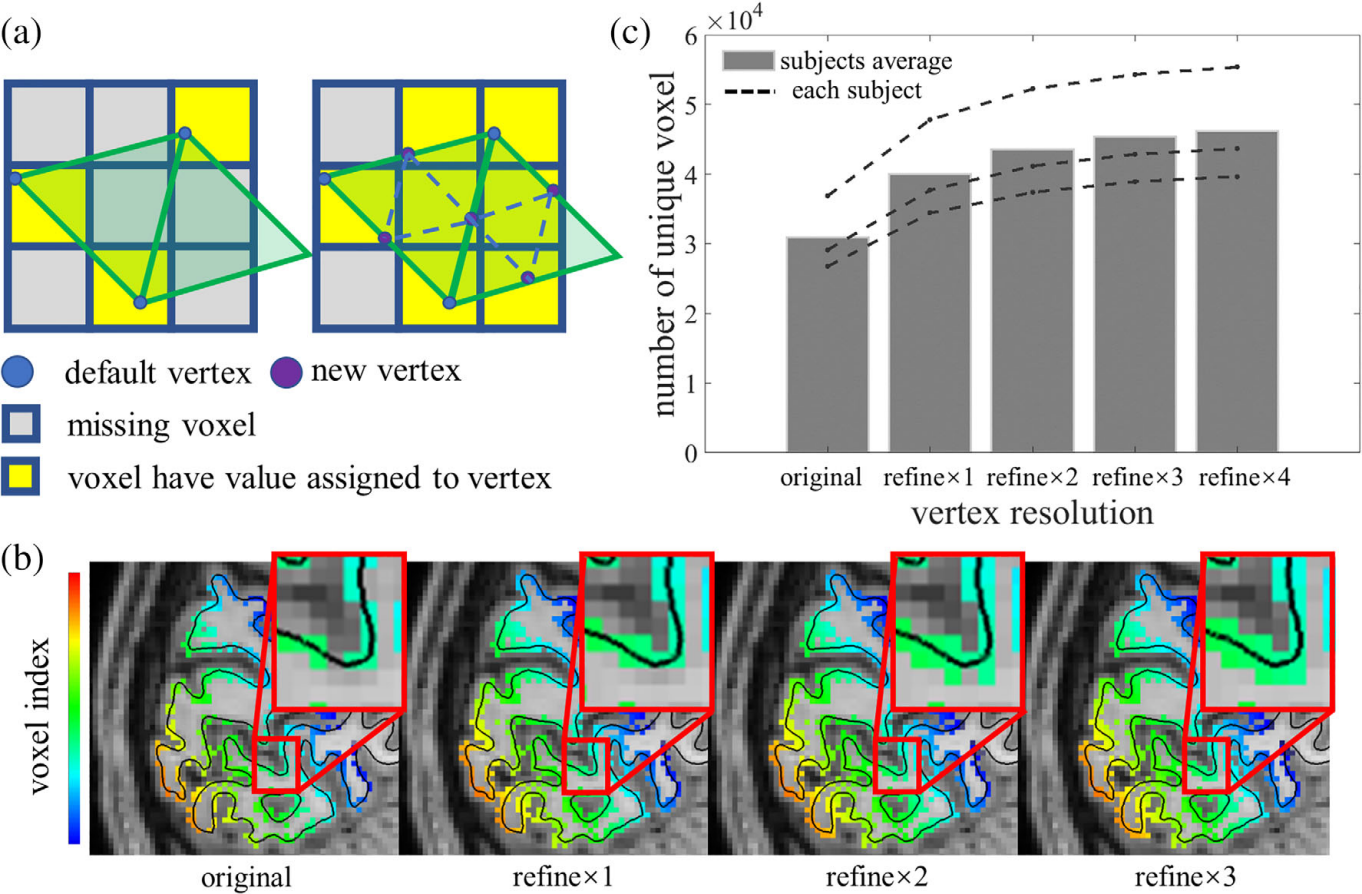
Effect of surface mesh refinement on reducing the number of missed voxels. (a) Schematic of volume‐to‐surface projection. Squares indicate the voxel in volume space. Yellow squares in represent voxels assigned to a mesh vertex. Gray squares represent missed voxel that were not projected onto any mesh vertex due to the coarse vertex spacing. Triangles represent the faces of the surface mesh. Blue dots indicate vertices in the original surface. Purple dots indicate vertices added through surface refinement. As vertices are added to the surface during refinement, a larger number of voxels are assigned to a vertex, which reduces the number of missing voxels. (b) Voxels missing when using the original surface that were captured with increasing iterations of surface refinement. The zoomed‐in views show an example surface (black contour) within the calcarine sulcus. Color indicates the unique voxel index. As the surface mesh is refined, more unique voxels are included in the surface projection. (c) The number of unique fMRI voxels as the surface mesh is progressively refined. With each iteration of refinement, steadily more unique voxels are included, with a plateau emerging after four refinement iterations. Dashed lines represent values for each individual subject (*N* = 3); bars show the average across subjects

As depicted in the schematic in Figure 4a, the mesh refinement scheme used here adds new vertices to the triangle edge mid‐points, and as the number and density of the vertices increases more unique voxels are retained during surface projection. This schematic also shows how refining the surface mesh can not only help reduce the number of missing voxels, but can also help minimize small‐scale spatial distortions or displacements that are incurred when projecting voxel values to vertices that are spatially distant from the corresponding voxel centroid. If the distance between neighboring voxels is smaller than the distance between neighboring vertex, there will be a chance that some voxels will be missed and not projected onto any vertex. As expected, Figure 4b shows, for an example subject, how the number of unique voxels projected onto the surface increases with increasing density of vertices as the mesh is iteratively refined from one to three steps. This evaluation was performed on the gray/white matter boundary surface generated by FreeSurfer from standard 1‐mm anatomical data, whose cross‐section with the image slice is represented by the black contour, because this cortical surface is more commonly used to preserve spatial specificity in BOLD fMRI studies. In order to visualize the unique voxels projected onto the surface, here we visualized the voxel index projected onto the surface as a mask in volume space. As can be seen in the zoomed‐in view, progressively more unique voxels near the gray/white matter boundary surface are retained (i.e., fewer voxels are lost) as the surface mesh is refined. Figure 4c shows quantitatively the number of unique voxels captured during surface projection with increasingly refined surface meshes, and demonstrates an increase in unique voxel count with increasing vertex resolution that is in line with what can be seen qualitatively in Figure 4b. Perhaps surprisingly, the number of unique voxels only reaches a plateau after three iterations of refinement for this example 1‐mm resolution volumetric fMRI data, suggesting that further steps of refinement may be necessary for even higher resolution fMRI data.

### 3.5 Evaluation of spatial blur on measured fMRI data with interdigitated spatial organizations

Next, in order to evaluate how do the preprocessing strategies impact the spatial blur of final activation pattern, we also took advantage of a favorable property of our fMRI data—the interdigitation of V2 thin and thick stripes—to test whether strategies that reduce blurring result in smaller spatial overlap between these stripes. In our data, the fine‐scale color selectivity “thin” stripes and disparity selectivity “thick” stripes in V2 were imaged in different experimental sessions taking place on different days. Then the overlap of the independently derived activation patterns was evaluated. In this way, less spatial blur induced by preprocessing will manifest as less overlap between these interdigitated stripe patterns.

**FIGURE 5:**
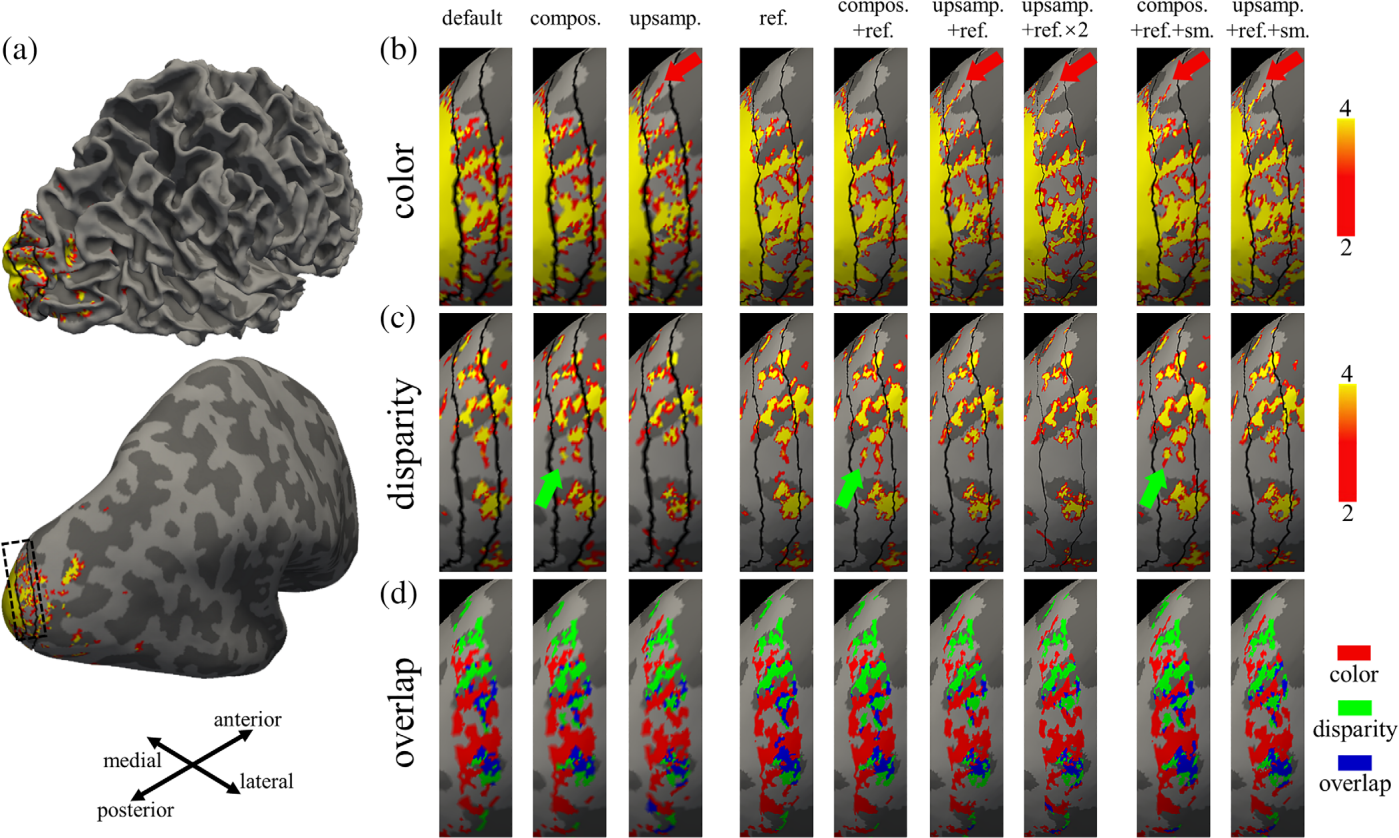
Evaluation of spatial blurring using functionally segregated, spatially interdigitated columnar sub‐systems. Activation maps and corresponding overlap maps of the V2 “thick” and “thin” stripe systems for an example subject. Each panel shows an activation overlay on the cortical surface, both before inflation and after inflation. Dark gray underlay indicates sulci; light gray indicates gyri. (a) Whole‐brain view showing the region of interest (indicated by dash rectangle) shown in panels (b)–(d), visualized from a lateral‐posterior viewpoint. (b and c) Qualitative evaluation of color selective “thin” stripes and disparity selective “thick” stripes, respectively. Each row shows thresholded (*p* <  0.001) activation maps of color selective thin‐stripes or disparity selective thick‐stripes, and each column shows the activation map resulting from a different preprocessing strategy. Color bar represents −log(*p*) of the activation. Black lines overlaid on each activation map indicate the borders of V1/V2 and V2/V3. (d) Overlap between the “thin” and “thick” stripes within V2. Red represents vertices identified as exclusively within the “thin” stripe; green represents vertices identified as exclusively within the “thick” stripe; blue represents vertices identified as both “thin” and “thick” stripes, that is, the regions of overlapping stripe sub‐systems. Red and green arrows indicate examples of fine‐scale activation pattern details retained by strategies like transformation composing, volume upsampling, and surface refining for “thin” (red) and “thick” (green) stripes respectively. “Default”, Default preprocessing strategy with default surface vertex spacing and sequential spatial transformations. “Compos”, Strategy of composing spatial transformations. “Upsamp”, Strategy of volume upsampling. “Ref”, Strategy of surface mesh refinement (one iteration). “Ref × 2”, Strategy of surface mesh refinement (two iterations)

Both stripes plus the overlap between the two are shown for one example subject visualized on the subject's cortical surface reconstruction in Figure 5 across different preprocessing strategies. We restricted the evaluation of overlap to activation within V2 because the interdigitation of these sub‐systems is only well established within V2. All of the preprocessing strategies tested here successfully imaged the stripes and by inspection, the “thin” and “thick” stripe interdigitated pattern looks qualitatively similar across the various strategies. However, fine details of the activation pattern do depend on the preprocessing approach taken.

Specifically, the first column in Figure 5b presents the activation maps resulting from the default preprocessing strategy. The following two columns are results from preprocessing strategies with composed spatial transformations (including surface projection) and with sequential spatial transformations (including surface projection) applied to the upsampled fMRI volume, respectively. Note that in order to isolate the impact of spatial blur reduction from other aspects that could affect the appearance of the maps, like registration accuracy (which may be affected by, e.g., upsampling the volumetric data), we applied the same spatial transformations and used the same functional‐to‐anatomical registration for all of the preprocessing schemes evaluated here. Therefore, any differences can be attributed to the effects of interpolation. In agreement with our conclusions from the synthetic data tests described above, more high‐frequency features of the activation map can be observed in the patterns generated using the volume upsampling strategy.

The second group of activation maps (columns 4–7) is arranged to show the effect of surface refinement. The results of surface refinement combined with the default preprocessing approach and results from surface refinement combined with composed transformations and volume upsampling are shown in columns 4–7, respectively. Qualitatively, surface refinement leads to an improvement in the depiction of the stripe patterns, where the composed transformation strategy combined with surface refinement helps to improve the continuity within the stripes, and several fine‐scale features appear (indicated by red and green arrows).

The third group of activation maps corresponds to the results from adding anatomically‐informed intracortical smoothing along the radial (or columnar) direction to test whether increased SNR can be achieved without losing fine‐scale detail in the columnar patterns. Notably, the intracortical smoothing does not appear to reduce the resolution of the activation pattern but it does reveal some fine‐scale features of the stripe patterns indicated by red and green arrows in columns 8 and 9.

**FIGURE 6:**
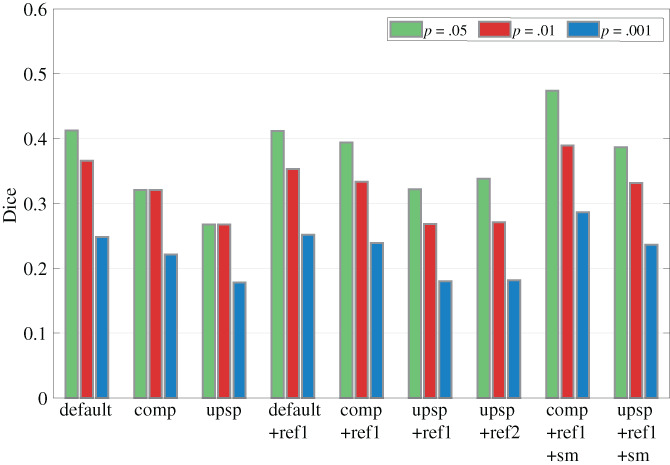
Quantitative evaluation of overlap between “thin” and “thick” stripes using the Dice coefficient. Each bar group shows overlap coefficient from different strategies. Each color represents the overlap calculated with a different statistical threshold to binarize the activation maps. Less overlap between “thin” and “thick” stripes indicates less spatial blur. “Default”, Default preprocessing strategy with default surface vertex spacing and sequential spatial transformations. “Compos”, Strategy of composing spatial transformations. “Upsamp”, Strategy of volume upsampling. “Ref”, Strategy of surface mesh refinement (one iteration). “Ref × 2”, Strategy of surface mesh refinement (two iterations)

To support this qualitative evaluation with a quantitative assessment, we also calculated the Dice coefficient to measure the extent of overlap of the interdigitated stripe sub‐systems across the different preprocessing strategies tested for all three subjects. The Dice coefficient is presumed to be lower in data with less spatial blur. Because overlap of the thresholded activation maps will be influenced by the choice of threshold, we calculated the Dice coefficient with several thresholds (i.e., 0.05, 0.01, and 0.001). As expected, overlap increases with lower threshold in general. Consistent with our qualitative evaluation, both the composed transformation and volume upsampling strategies lead to a decreased overlap (Figure 6). This suggests that these strategies both reduce the amount of spatial blur, as expected. Interestingly, thresholds of 0.05 and 0.01 both result in a nearly identical Dice coefficient for both the composed transformation and volume upsampling strategies, suggesting that these strategies probably induce less false positives. Surface refinement has a small impact on the overlap coefficient, which implies that the trilinear interpolation used for volume‐to‐surface projection helped to reduce voxel losses during projection, which led to a smaller effect of refinement. While volume upsampling resulted in the most favorable overlap, surface smoothing slightly worsened the overlap, which indicates either that some spatial smoothing in the tangential direction may have occurred or that the increased number of above‐threshold vertices caused an increase in overlap. Nevertheless, combining the surface refinement with intracortical smoothing did result in a net increase in the number of activated vertices, and the avoidance of resolution losses due to upsampling seems to have partially compensated for the loss of resolution due to explicit intracortical smoothing such that the resulting overlap is similar to the default preprocessing scheme—while providing higher details and more fine‐scale features seen in the columnar patterns.

### 3.6 Higher resolution increases motion estimation accuracy

**FIGURE 7:**
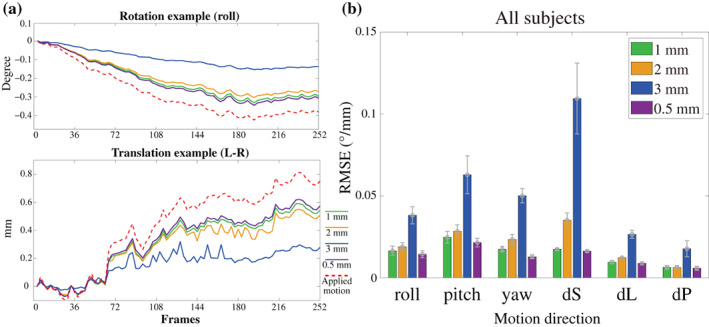
Effect of imaging resolution on motion estimation accuracy. (a) Example motion traces used for generating synthetic data, computed from a run of our fMRI data; only one rotation direction and one transformation direction are shown. (b) The RMSE between the applied and the estimated motion parameters at 1, 2, 3, and 0.5 mm voxel sizes. Red dash lines in (a) indicate the applied motion parameters used as ground truth. Green, orange, blue, and purple colors indicate 1, 2, 3, and 0.5 mm voxel sizes. RMSE, root‐mean‐square error; Roll, pitch, and yaw, rotation about the I–S axis, R–L, axis, and A–P axis; dS, dL, and dP, Displacement in the superior direction, left direction, and posterior direction

There are other aspects of preprocessing that may not influence the spatial blur directly but are indeed beneficial to the spatial accuracy of the data. In the previous section, we showed how volume upsampling can improve spatial specificity. However, volume upsampling can also potentially improve motion estimation and correction accuracy. To quantify the impact of voxel grid spacing on motion estimation accuracy, we performed a series of tests on synthetic 4D data with known motion. As shown in Figure 7, the difference between the true motion parameters and the estimated motion parameters decreases after upsampling the volume, indicating that a finer grid spacing can increase motion estimation accuracy. Conversely, using a coarser grid spacing decreased motion estimation accuracy, with larger voxels further underestimating the motion. This indicates that upsampling the image volume prior to estimating motion can be beneficial, and then when correcting the motion with the resulting spatial transformations also provides benefits indicated above in terms of reducing resolution losses due to interpolation.

Next, we tested whether an improvement in motion estimation accuracy can be similarly achieved by acquiring data with higher resolution. To do this, we again generated synthetic data with known motion, but here we generated the data on various voxel grid spacings. These results are presented in Figure S1, which also show higher accuracy of motion estimation from the finer grid spacing and lower accuracy from the coarser grid spacing. While, in general, it is not always possible to increase fMRI resolution (due to hardware restrictions and limited SNR), these results suggest that, perhaps counterintuitively, while higher‐resolution data may be viewed as more vulnerable to small motion, in fact, smaller voxels may partly compensate for this by improving the accuracy with which the motion is estimated.

### 3.7 Spatial varying resolution caused by geometric distortion correction

**FIGURE 8:**
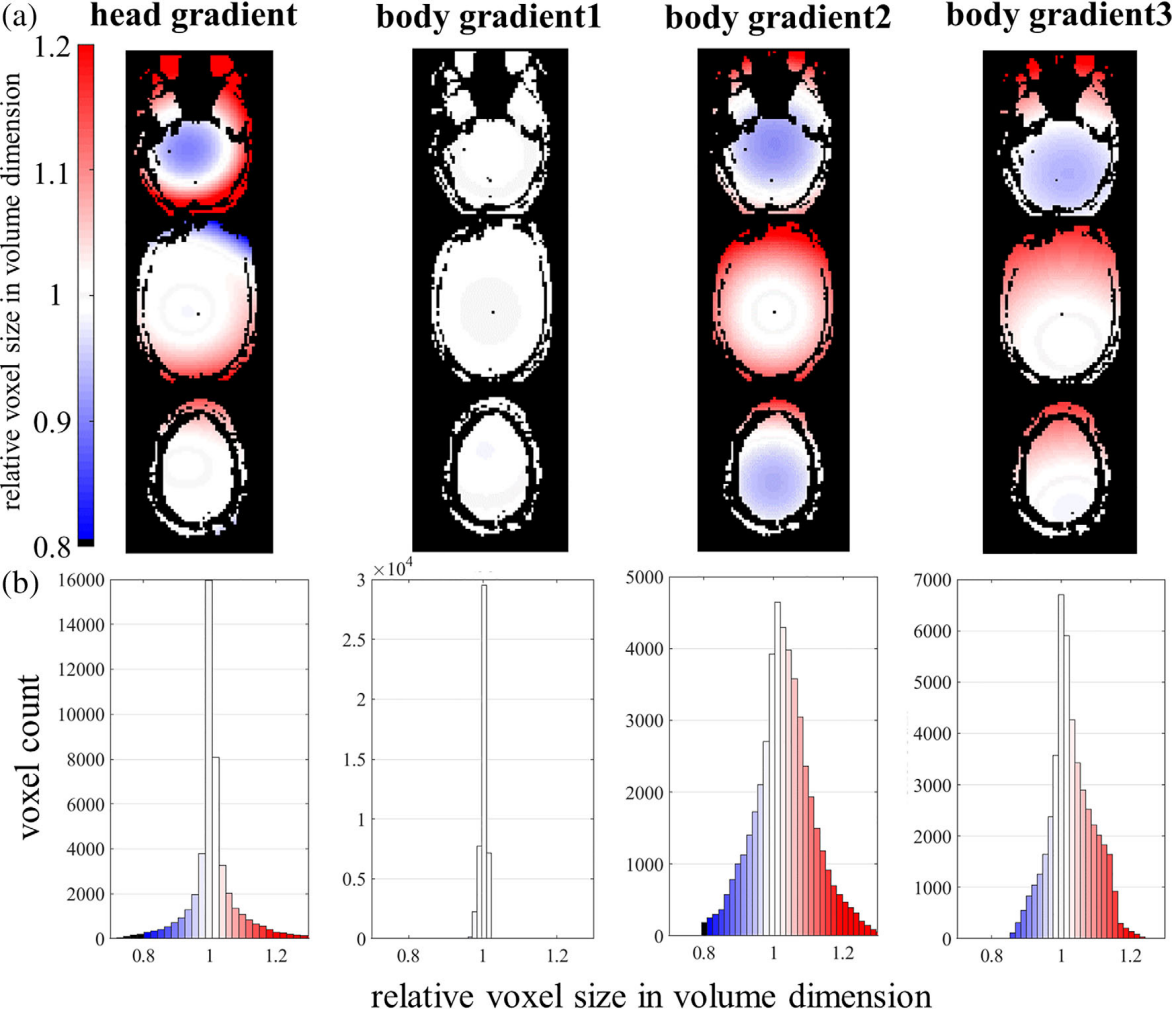
Spatial nonuniformity of resolution induced by geometric distortion due to gradient nonlinearity. Voxel size varies smoothly across brain regions due to geometric expansion and compression from gradient nonlinearity, which varies with the design of the gradient coil. Examples from whole‐brain axial slices (a) and histograms (b) show the spatial distribution of relative voxel size in volume dimension. Color range from blue to red indicates smaller to larger true voxel sizes

A final source of resolution loss that we will consider is geometric distortion. While distortion is well‐known to cause spatial errors by displacing voxels, this nonlinear warping of the data also can expand or compress voxel sizes, leading to another form of spatially varying resolution across brain regions. A simple means to evaluate true imaging resolution is by computing the voxel compression/expansion embodied by Jacobian determinant of the deformation. As a simple example of a common form of spatial distortion, here we evaluated voxel compression/expansion imparted by gradient nonlinearity of several modern gradient coil sets. As shown in Figure 8, gradient nonlinearity causes the effective resolution to vary spatially. The pattern of spatially varying voxel size of course varies with the gradient coil design, as the nonlinearity is a function of the gradient coil winding. The exact pattern of this spatially varying resolution will also depend on the location of the brain relative to the gradient coil (i.e., the subject's head position relative to isocenter).

Because the head location in our dataset is near to the isocenter, and because the gradient coil used in our fMRI data acquisition is among the most linear of all coils tested, we quantified the influence of gradient coil nonlinearity in our fMRI and found it to be negligible in our region of interest, the visual cortex (shown in Figure S2), therefore, we chose to not perform any gradient nonlinearity correction on our data since it was not necessary. Nevertheless, because gradient nonlinearity is pronounced in high‐performance gradient coil designs, we do recommend that this source of spatially varying voxel size—which is straightforward to compute—should at least be evaluated in high‐resolution fMRI studies (if not corrected), as it may impact the interpretation of the results.

## 4 DISCUSSION

Here we have quantified the effects of various fMRI preprocessing strategies on the effective resolution of the data. Because high‐resolution fMRI studies are often performed at the limit of achievable imaging resolution, any form of resolution loss imparted during data analysis should be minimized. Our study, using both synthetic and real data, shows that the level of blurring will depend on several analysis choices. One major source of resolution loss is that incurred by interpolation, and we evaluated several strategies to mitigate this, including the composition of spatial transformations and surface projection, as well as a simple but relatively underappreciated approach consisting of upsampling the fMRI volume. Relevant to the increasing number of high‐resolution fMRI studies utilizing surface‐based analyses, we also evaluated the effects of surface mesh refinement and interpolation algorithm. As expected, and as noted above, spatial transformation composition, volume upsampling, and surface mesh refinement do help to retain the spatial resolution and, as a consequence, the fine‐scale features. Surprisingly, the simple approach of volume upsampling performs as well as transformation composing.

Using synthetic white noise data, we showed that preprocessing can result in a loss of resolution, which in turn will limit the detectability of fine‐scale structures. Moreover, we showed that the induced spatial blur effect is nonuniform over the brain and therefore can be a source of spatially varying resolution or detection bias. The spatial pattern of this resolution loss will depend on factors such as the pattern of head motion, which will vary across runs and subjects, and on factors such as the pattern of gradient nonlinearity, which will remain constant. While several previous studies (referenced below) have pointed to several of these effects, and some of our observations of resolution loss were expected, we also provide a simple framework for evaluating resolution based on applying the preprocessing steps to white noise data, which can be helpful for comparing different strategies and quantifying the resulting blur. This approach is also amenable to complicated preprocessing steps for which a closed‐form analytic expression for the induced blur is not readily available. The consequences of these strategies were demonstrated here by applying them to mapping fine‐scale features of functional architecture, the V2 stripe system of the visual cortex, for which there is a strong prior regarding the spatial pattern of activation that can allow us to evaluate these various methods in a more concrete way. In addition, we have presented several quantification methods, including novel evaluations of motion parameter estimation accuracy and missing voxels during surface projection, to provide a deeper understanding of the advantages and disadvantages of these strategies applied to high‐resolution fMRI studies.

### 4.1 Overview of previous studies regarding these problems

The strategy of mathematically composing the multiple spatial transformations applied to the data has been proposed by previous studies as a means to reduce the number of interpolation steps and thereby minimize resolution losses (Esteban et al., 2019; Glasser et al., 2013). In those studies, both rigid and nonrigid (i.e., nonlinear) transformations—including motion correction, distortion correction, functional‐to‐anatomical registration, and spatial normalization to a common 3D atlas space—were composed in volume space. Our study also considered the step of composing the projection from volume space to surface space. Here the motion correction transformation and the functional‐to‐anatomical transformation were composed so that the volumetric fMRI data could be projected directly onto the surface mesh without any volume interpolation. Thus, the only interpolation occurs at the stage of projecting the voxel data onto the surface mesh vertices.

A similar idea has been recently proposed (Huang et al., 2020) in which motion correction is applied by aligning each fMRI frame directly to the anatomical image, rather than by aligning each fMRI frame to a reference frame. This approach was motivated in part by the availability of powerful “boundary‐based registration” methods that accurately align the fMRI data sampling the cortex to the cortical surface, and successfully removes one step of resampling and interpolation of the functional data. The difference between their approach and ours is that they estimate the motion through anatomical registration, whereas our procedure, in order to be more directly comparable to existing approaches, calculated the motion estimation by aligning each fMRI frame to a reference frame and then registered the reference frame to the anatomical data using boundary‐based registration. While there are advantages and disadvantages to both approaches, we expect the accuracy of the motion estimation to be comparable in general, and the direct approach of motion correction through registration may perform better in studies using a restricted FOV or a small number of slices for the fMRI acquisition where the vulnerability to out‐of‐view or through‐slice motion is high.

Moreover, the current study focused on evaluating to what extent the preprocessing steps influence the spatial blur of fMRI data. While SNR is an important metric for evaluating fMRI data quality, blurring can of course increase SNR, and so SNR gains often come at the cost of resolution losses. Here our focus was on minimizing resolution losses, while Huang et al. focused on improving SNR and thus made recommendations on preprocessing approaches that reduced temporal variability in the fMRI data (Huang et al., 2020). Future work may consider both quality metrics to establish a given desired balance between resolution and SNR (i.e., between specificity and sensitivity).

Volume upsampling, being another possible strategy to reduce the spatial blur caused by preprocessing, was also evaluated and compared to the strategy of composing spatial transformations. Perhaps surprisingly, we found that simple volume upsampling was also effective at reducing blur, and often performed better than the more sophisticated approach of the mathematical composition of transformations. We note, however, that one limitation of our study is that we considered only a small number of transformation steps, and that as the number of transformations applied to the data increases, the performance of transformation composition relative to volume upsampling is expected to increase; here only two transformation/projection steps were considered, but for studies using three or more such transformations are likely to see that transformation composition will result in less blurring than volume upsampling.

Transformation composition does come with practical challenges, as it requires that transformations from potentially different software packages—or even from different tools from a single package—have compatible conventions and formats, and often translating between conventions can be cumbersome and error prone. Although newly developed wrapper packages like fMRIPrep (Esteban et al., 2019) sought to integrate several common spatial transformation routines from the most widely used packages, it is difficult to stay fully up‐to‐date with the many new and updated packages and routines available. Therefore, volume upsampling may be an attractive option for many users due to its simplicity. The main downside to this approach is that it requires increasing data size (e.g., by a factor of 8 to upsample by the smallest integer factor of 2), which creates potential practical issues with data storage capacity, and it increases the computation burden, which creates potential practical issues with computer memory and processing time.

Note that upsampling does not lead to changes in the number of comparisons. That is, upsampling simply duplicates the number of samples without introducing any new values. For example, when a single voxel is divided into eight during volume upsampling using nearest‐neighbor interpolation, the number of degrees of freedom in the data and thus the number of comparisons does not change. This is because, after upsampling, the new datapoints are not independent. So, the number of comparisons is still equal to the original number of voxels. Also, smoothing induced by interpolation or other steps of preprocessing can decrease the spatial degrees of freedom, and so spatial correlations of the fMRI data after preprocessing (e.g., using the residuals) are often measured to inform multiple comparisons corrections (Hagler et al., 2006).

While here we have demonstrated how reducing blurring through appropriate choice of preprocessing steps aids in fMRI studies of cortical columns, reducing unwanted blurring should also yield benefits in other applications of high‐resolution fMRI such as investigations of small subcortical nuclei and studies of cortical layers. Previous laminar fMRI studies have made observations similar to ours regarding analysis steps that are specific laminar fMRI: it has been shown that upsampling in the laminar direction—or using a “finer laminar grid” composed of far more depths sampled compared with number of voxels that span cortical thickness—can provide better depictions of the laminar profile (Huber et al., 2017; Huber et al., 2018; Polimeni et al., 2018). For example, generating profiles with up to 20 depths can be advantageous, even when using voxels that are barely sub‐millimeter in size.

### 4.2 Towards a reliable strategy for quantifying spatial blur

Often the visual appearance of the imaging data can be used to assess smoothness, but this is subjective and not reliable. In order to quantify the effect of spatial blur induced during preprocessing, we subjected preprocessing procedures to synthetic i.i.d. white‐noise time series data matched to the voxel grid spacing of the functional data, resulting in filtered noise. This is similar to Monte Carlo approaches used to assess spatial correlations in the fMRI data for cluster size estimation used in multiple comparisons correction approaches (Hagler et al., 2006). In contrast to methods that evaluate spatial smoothness from the fMRI data directly, such as residual‐based estimation (Kiebel et al., 1999) which assumes the residuals are white noise, we force the noise in the input data to be white. Because methods based on residuals also do not account for meaningful short‐ and long‐range spatial or temporal correlations in the fMRI due to structured physiological noise or due to functional connectivity (Eklund et al., 2016; Wald & Polimeni, 2017), they may not be universally applicable. Our approach, therefore, may be better suited to estimating the smoothing imposed in the data due to preprocessing, since it avoids statistical assumptions on the fMRI data that may not hold for a given dataset.

Furthermore, our method provides a measure of voxel‐wise blurring effects which is different from the conventional approaches that attempt to estimate spatial blur by examining correlations in the data (or in the residuals) along the three dimensions of the voxel grid and inferring a 3D smoothing kernel from this. As we demonstrated above, because the induced spatial blur is nonuniform across the brain, our voxel‐wise blur estimation could potentially be used for region‐specific multiple comparison correction, or for providing a “spatial error bar” along with the activation map so that the results can be better interpreted in light of the regional patterns of spatially varying resolution.

### 4.3 Potential influences of underlying image features on blurring when using high‐order interpolation

One consideration of our white noise approach is that it does not account for the possibility that the smoothing induced by preprocessing may itself be influenced by the underlying image content. For example, higher‐order data interpolation forms nonlinear combinations of sampled data to estimate new data, and the weighting depends on the intensities of the sampled data. Therefore, applying these interpolation methods to zero‐mean noise data will not produce the same interpolation effects seen when applying them to nonzero‐mean imaging data, and so our estimated smoothing based on zero‐mean noise may be rough approximation only.

To address this, we also applied zero‐mean noise to a high‐SNR frame of fMRI data (generated by averaging several frames), subjected both the high‐SNR frame and the frame with added noise to several preprocessing steps that used high‐order interpolation, subtracted the two resulting volumes, and re‐characterized the resulting TSTD and corresponding FWHM, and the differences in the results of this new test and the results reported above were negligible. Nevertheless, because some of these effects may depend on the specifics of the underlying image structure or anatomy, while our results are likely to be valid in general, future studies may re‐visit these characterizations on specific cases of interest, such as the potential for different levels of blurring within the cortex and at the border of the cortex (due to potential edge enhancement effects).

### 4.4 Disadvantages of nearest‐neighbor interpolation

Although we demonstrated through simulations based on synthetic noise data that nearest‐neighbor interpolation may be advantageous since it induces the smallest spatial blur (Figures 2 and 3), we chose not to use nearest‐neighbor interpolation in our V2 stripe analysis, rather we opted to use trilinear interpolation. Our reasoning behind this decision to not use nearest‐neighbor interpolation is that it also causes displacement and local distortion artifacts, both in the context of volume transformations and surface projections.

Nearest‐neighbor interpolation applied to when transforming volumetric data is known to does not correctly account for subvoxel displacements (Grootoonk et al., 2000) because it is a nonlinear interpolation—small displacements relative to the voxel grid may cause no changes in intensity after interpolation, and if the magnitude of displacement increases steadily there can be sudden jumps in intensity after interpolation.

Nearest‐neighbor interpolation applied to volume‐to‐surface projection when using coarse grid spacing on surface mesh relative to a dense volume grid can cause displacement artifacts (i.e., fine‐scale distortion) and missing voxels (Figure 4). This can lead to artifactual truncated appearance of the data projected onto the surface; this manifests as an aliased appearance of the activation pattern and artifactual edges, which may give misleading fine‐scale structure in the activation pattern. Nevertheless, we note that nearest‐neighbor surface interpolation can be beneficial provided that the surface mesh spacing is sufficiently fine relative to the volume resolution.

### 4.5 Other methods exist for representing voxel data on surface

Our results show that surface refinement can help decrease the number of missing fMRI voxels when projecting data onto cortical surfaces. However, our tests demonstrate that the number of unique voxels does not plateau until three iterations of refinement have been performed. If we were to require that all fMRI voxels intersecting the cortex are included in the projection, an even denser vertex mesh might be needed, however, each step of refinement dramatically increases the computational load of the analysis while providing diminishing returns. We note that the number and density of vertices are dependent upon the algorithm used to reconstruct the surface mesh, and in the method used by FreeSurfer these are a function of the voxel grid used in the anatomical data, which in our case was a 1‐mm MPRAGE. As higher‐resolution anatomical data, which have been shown to provide improved surface reconstruction accuracy, become more common, so will higher density surfaces, which will require less refinement.

To preserve all fMRI voxels in surface‐based analyses regardless of the density of surface vertices, Gao et al. suggested a pixel‐based method for projecting onto surfaces (Gao et al., 2015). This pixel‐based algorithm directly renders the voxel coordinates on the surface, such that each fMRI voxel is always represented on the surface regardless of the vertex spacing, thereby eliminating the need to upsample the surface mesh. This also removes the need to interpolate the voxel data when projected onto the surface mesh, and avoids local displacements or distortions of the fMRI data, which helps to preserve the spatial specificity and accuracy. While this rendering approach is promising for visualization, it may not be amenable to other forms of surface‐based analysis where further processing of the fMRI data along with the surface is necessary, such as surface‐based intracortical smoothing.

Although we demonstrated how relevant fMRI voxels may be dropped from analysis when projecting onto cortical surface meshes, the number of missing voxels reported was the average number across the cortex evaluated using multiple datasets. In other words, dropped voxels do not occur uniformly across the cortex. This is partially because the irregular triangular surface mesh consists of vertices with nonuniform spacing (i.e., the distances between neighboring vertices varies across the mesh; Kay et al., 2019; Kemper et al., 2018). An approach using regularly spaced quadrilateral mesh can also be an alternative means to represent voxel data on the surface while maintaining a uniform sampling of the voxel data on the surface (Kemper et al., 2018; Zimmermann et al., 2011). In this approach, each fMRI in the regular quadrilateral grid is always surrounded by four neighboring vertices at a nearly constant distance forming 90° angles between edges. This representation requires a fine spacing in order to maintain regular quadrilaterals (i.e., a square grid) given the nonzero Gaussian curvature of the cortex. Further studies are needed to evaluate the advantages and disadvantages of these two approaches, however, they both acknowledge and address the generally underappreciated problem of how best to represent volumetric imaging data on surface representations that minimizes data loss and fine‐scale distortions incurred when projecting these data onto the cortical surface.

### 4.6 Motion estimation accuracy

Along with our evaluations of spatial blurring, we evaluated the spatial accuracy of motion estimation and correction and tested how changes in voxel size—imposed either during preprocessing or during acquisition—affected this accuracy. The first test was conducted by applying measured motion parameters to generate a 1‐mm fMRI volume with known motion, then resampling this volume to 0.5, 2, and 3 mm grids to emulate how volume resampling during preprocessing might impact motion estimation accuracy. The second test was conducted by applying measured motion parameters to 0.5, 1, 2, and 3 mm grids to emulate how acquiring data at varying resolutions might impact motion estimation accuracy. Both sets of results indicated that smaller voxel sizes could improve the accuracy of rigid‐body motion estimation.

Interestingly, the second test suggested that, although the expectation would be that high‐resolution acquisitions would have stricter requirements for subject motion, since a sub‐millimeter acquisition would be less tolerant to sub‐millimeter motion, perhaps counter‐intuitively motion estimation is more accurate with smaller voxel sizes, and so this increased accuracy may help compensate for this increased vulnerability to motion. Higher‐resolution fMRI acquisitions seemingly have several unexpected benefits, since it has also been demonstrated that smaller voxels also help to reduce partial volume effects and can thereby help reduce physiological noise contamination and signal dilution effects from neighboring tissues (Blazejewska et al., 2019). Indeed, smaller voxels will similarly reduce challenging sub‐voxel motion and will also reduce the associated blurring, as shown above.

### 4.7 Effects of nonrigid transformations and distortions

While most of our evaluations focused on the effects of rigid transformations on spatial resolution imparted during preprocessing, we also briefly considered the effects of nonrigid transformations and distortions. Just as rigid transformations lead to losses due to interpolation, nonrigid or nonlinear transformations similarly lead to spatial resolution losses due to interpolation, and these effects have been characterized previously (Glasser et al., 2013; Polimeni et al., 2018). In this way, nonrigid transformations are similar to rigid transformations, and induce spatially varying resolution losses that are a function of the voxel grid, the interpolation method, and the transformation itself.

Beyond this, we also consider how nonlinear distortions can manifest as a spatially varying voxel size that is present at the time of data acquisition. While distortion can potentially be corrected, this spatially varying imaging resolution cannot be corrected in postprocessing, therefore a method to quantify this nonuniform resolution can be helpful for interpreting the data. Here we propose to use the Jacobian determinant of the nonlinear transformation, which has long been used in the context of voxel‐based morphometry (VBM) to reveal morphometrical differences in anatomical images by computing nonlinear transformations between pairs of images (or between an image and a template space) then evaluating the Jacobian determinant of that transformation as a proxy for regional, relative expansion or compression of the brain between the two images. This local expansion or compression of the true voxel size occurs whenever the image data are a warped version of the true anatomy. The most commonly considered form of this geometric distortion is that induced by susceptibility gradients in EPI data, and can be quantified by measuring B_0_ inhomogeneity.

Another perhaps simpler form of geometric distortion is that caused by gradient coil nonlinearity. However, spatially varying changes in true voxel size that accompany these forms of geometric distortion are less appreciated in fMRI studies. Our study, through evaluating gradient nonlinearity distortion across different gradient coil designs, illustrated that the effective resolution can vary from about 20% smaller to 20% larger than the original voxel size. While the range of voxel size scaling will depend on the gradient coil and on the position of the head relative to isocenter, this form of spatially nonuniform resolution should be considered in fMRI applications where the spatial scale of the targeted activation pattern is relevant, or when hypotheses are being tested that require a specific resolution. The impact of susceptibility‐gradient‐induced distortion on voxel size could also be readily calculated given a map of B_0_ inhomogeneity, although estimating an accurate B_0_ map can be challenging in practice (Hutton et al., 2002; Irfanoglu et al., 2015).

If the true voxel size differs substantially from the nominal voxel size, this may lead to incorrect conclusions if this is not taken into account. Of note, this effect of distortion on resolution not only expands the voxel size, leading to undesired resolution losses, but can also compress the voxel size, leading to beneficial resolution gains. It may be possible to take advantage of this effect to achieve higher effective image resolution in certain regions of the brain, if the pattern of distortion is accurately known in advance.

The Jacobian determinant is not only helpful for quantifying resolution losses imparted during preprocessing but also helpful for quantifying the resolution of the acquired data. It is also possible then that this spatially varying voxel size will lead to a spatial varying SNR, as SNR is a function of voxel volume. Future studies may investigate to what extent the spatial varying voxel size contributes to spatial varying SNR using Jacobian determinant, although this is expected to be small compared to other factors that influence SNR across the brain such as the proximity to the receive coils.

### 4.8 Other aspects causing spatial blur in fMRI data

The present study focused on the spatial blur induced both during image data acquisition and during data preprocessing. We note that there are other MRI‐physics‐related sources of blur in fMRI data that were not addressed here, such as partial Fourier reconstruction (Zaretskaya et al., 2018) and T_2_* decay in EPI (Chaimow & Shmuel, 2016; Farzaneh et al., 1990), that also strongly influence the effective imaging resolution. Notably, T_2_* blur also varies substantially across the brain, and is further complicated by its dependence on voxel size and on the quality of the B_0_ shimming as well as other factors (Stockmann & Wald, 2018). In addition, there are also other biological sources, such as large vein effects, that can result in losses of spatial specificity including displacement artifacts that can distort activation maps (Kay et al., 2019; Olman et al., 2007).

### 4.9 Evaluation of spatial specificity using spatial interdigitated “thin” and “thick” stripes

Many studies have been dedicated to evaluate the spatial blur of fMRI data using a known feature of the activation pattern such as the retinotopic layout of neural activity in visual cortex (Polimeni et al., 2010), ocular dominance columns (Shmuel et al., 2010; Yacoub et al., 2007), and temporal frequency selectivity columns (Fracasso et al., 2021). Using the spatial interdigitation of left‐eye‐ and right‐eye‐dominant ocular dominance columns, a similar study has been performed to show the spatial blur effect induced by macrovascular contributions to the BOLD fMRI data (Yacoub et al., 2007). In these studies, differential maps of ocular dominance columns were generated from a single fMRI measurement, which enforced the two columnar sub‐systems to be spatially nonoverlapping, and so these data are not amenable to quantification of overlap between the two interdigitated columnar sub‐systems.

Our study employed activation maps generated from two independent datasets of two separate columnar structures, acquired across different experimental sessions, and evaluated the spatial blur effect using the overlap between these two separate maps. Another difference between the present study and previous studies is that they focused on the blurring effect of hemodynamics and large‐vein effects, while we focused on the effects of preprocessing strategies and image distortions.

We demonstrated that with different preprocessing strategies, the overlap between the functionally interdigitated “thin” and “thick” stripes can change. While all preprocessing strategies allowed us to detect both mostly nonoverlapping stripe patterns in all subjects, the two patterns were never observed to be purely distinct. This is to be expected, because, while we aimed to minimize blurring induced by preprocessing, the various forms of blurring mentioned above, including T_2_* blurring, distortion blurring, the limited resolution of the hemodynamics themselves, and the minimized‐but‐nonzero blur introduced through preprocessing will inevitably lead to some spatial smearing of the data. Furthermore, it is possible that these two systems are not perfectly interdigitated at the level of neuronal organization, and neurons within pale stripes may to some extent represent both color and disparity (Peterhans & Heydt, 1993), which may also contribute to overlap. Nevertheless, our results, both from synthetic and real data, consistently show a general trend in which less blurring induced by preprocessing leads to less overlap between these two largely interdigitated columnar sub‐systems.

### 4.10 Balance between spatial specificity and sensitivity

Here we have presented tools for assessing spatial accuracy in fMRI data. Because spatial resolution loss is often accompanied by increased SNR, often these two effects may counterbalance one another, especially for acquisitions that are SNR starved. For studies that lack sufficient statistical power, the improved SNR may be beneficial in some regions, and can even lead to an increased visibility of some features in the fMRI data. However, it is preferable to design experiments to have sufficient SNR so that these regional differences in smoothness do not introduce spatial detection biases (Viessmann & Polimeni, 2021). If more SNR is required, it is recommended to either require data with more runs or perform explicit anatomically informed smoothing. Regardless, it is important to know the spatial resolution—even if losses in resolution do provide occasional improvements—for proper interpretation of the data.

### 4.11 Relating reductions in smoothing to scan‐rescan reproducibility: Accuracy versus precision

As mentioned above, when seeking to reduce measurement error there is often a trade‐off between accuracy and precision, that is, there exists a bias‐variance trade‐off. The current study focuses on how to maintain high spatial accuracy by reducing unwanted forms of spatial blur. Spatial blurring is a form of systematic error which is reproducible. While scan–rescan reproducibility is an important metric for fMRI data quality, and is a measure of precision, reproducibility can be trivially increased by spatially smoothing the data, which of course comes at the cost of lower detectability of fine‐scale details in the activation pattern. Thus, reproducibility alone is not a sufficient measure of data quality, and indeed it can sometimes be misleading because does not always capture losses in spatial accuracy. Scan–rescan reproducibility is of course an important measure used to judge whether the activation pattern merely reflects spatial noise and thus the activation pattern is not meaningful, as random noise patterns are unlikely to be consistent across scans. However here, since we are mainly concerned with reducing spatial blurring, reproducibility is not a suitable metric to quantify whether spatial blurring has been successfully reduced through proper strategies for data preprocessing.

## 5 CONCLUSION

This study suggests multiple strategies to reduce the amount of spatial blur induced during fMRI data preprocessing. These strategies, including composing spatial transformations, volume upsampling, and surface mesh refinement, help capture fine‐scale details of the patterns of functional activation seen in our case through the detection of interdigitated columnar systems in the extrastriate visual cortex. We also note that not only is the level of spatial blurring dependent on preprocessing choices, but also it is nonuniform over the brain. Evaluation of the final resolution after preprocessing can help interpret the results in studies attempting to compare fine‐scale activation patterns between distant brain regions, characterize spatial details of columnar or laminar activation, or discover novel aspects of functional architecture that have yet to be seen with fMRI.

## CONFLICT OF INTEREST

The authors declare no competing interests.

## ETHICS STATEMENT

All experimental procedures were performed in accordance with the Massachusetts General Hospital approved Human Research protocol and federal guidelines.

## Supporting information


**Data S1** Supplementary Information

## Data Availability

The analysis software used in this study are available by request. The ethics protocol used for the data collection does not allow for data sharing, and so the data are not available at this time. The data and analysis code are available with the funding bodies requirements.
