# Supplementary material for: Critical factors in achieving fine‐scale functional MRI: Removing sources of inadvertent spatial smoothing
Source: Hum Brain Mapp. 2022 Apr 13;43(11):3311–31. doi: 10.1002/hbm.25867 (PMC9248309; doi:10.1002/hbm.25867)
Supplement: Supplementary file 1 — Data S1 Supplementary Information [file HBM-43-3311-s001.pdf]

Critical factors in achieving submillimeter-resolution functional MRI: reducing sources of inadvertent spatial smoothing

Jianbao Wang, Shahin Nasr, Anna Wang Roe, Jonathan R. Polimeni

Supplementary Figures

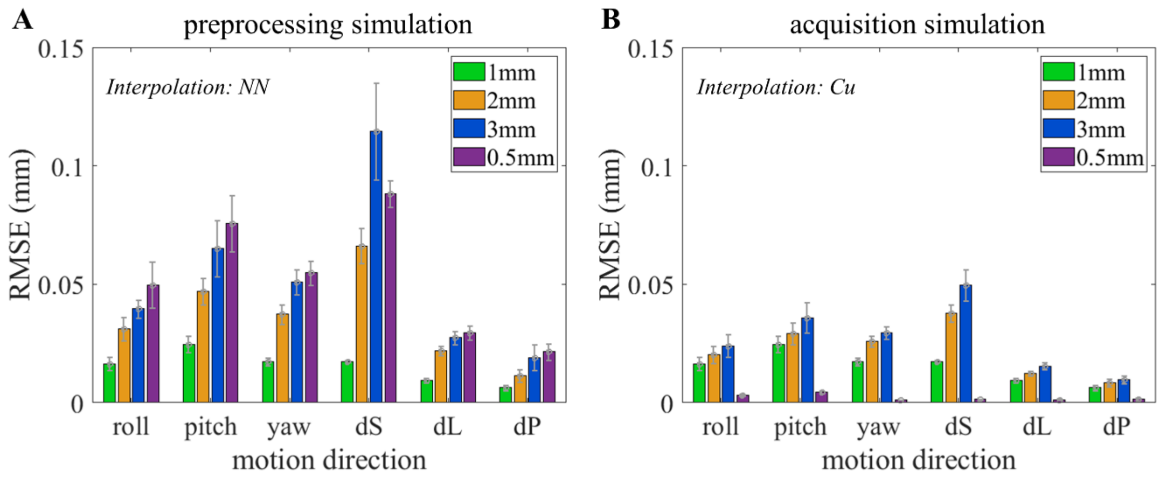

Fig. S1: Effect of imaging resolution on motion estimation accuracy. The RMSE between the applied and the estimated motion parameters at 1-mm, 2-mm, 3-mm and 0.5-mm voxel sizes. (A) Simulation quantifying the effect of volume resampled on motion estimation accuracy, to demonstrate whether this preprocessing step can help improve accuracy. Only interpolation method was changed to nearest-neighbor interpolation from cubic interpolation. (B) Simulation quantifying the effect of acquiring data with different voxel sizes on motion estimation accuracy, to demonstrate whether this change in the data

acquisition can help improve accuracy. The original 1-mm voxel data was resampled to 0.5 mm, 2 mm and 3mm voxel size followed by motion parameter applied. Green, orange, blue and purple colors indicate 1 mm, 2 mm, 3 mm and 0.5 mm voxel size data. Abbreviations: RMSE: root-mean-square error. Roll, pitch and yaw: rotation about the I–S axis, R–L axis and A–P axis. dS, dL and dP: displacement in the Superior direction, left direction and posterior direction.

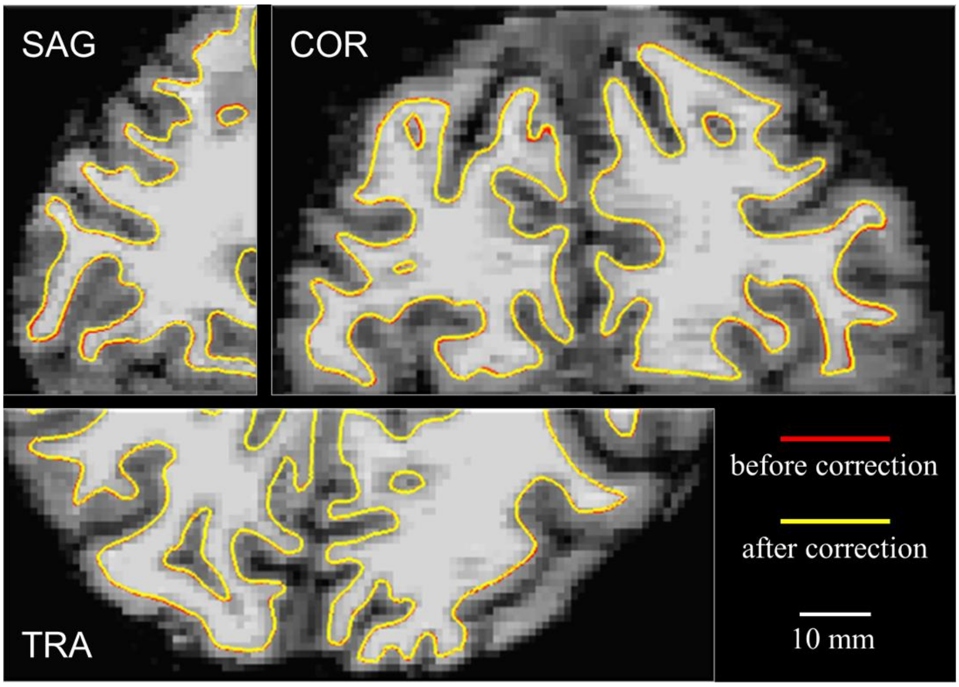

Fig. S2: Example shows negligible fMRI distortion caused by gradient nonlinearity distortion. Images show example of brain white/gray matter surface before (red lines) and after (yellow lines) gradient nonlinearity distortion correction. The gradient nonlinearity correction was applied both to the fMRI volumes and to the anatomical volume. The differences between surface before and after correction is small. Scale bar: 10 mm. Abbreviations: SAG: sagittal view. COR: coronal view. TRA: transverse view.

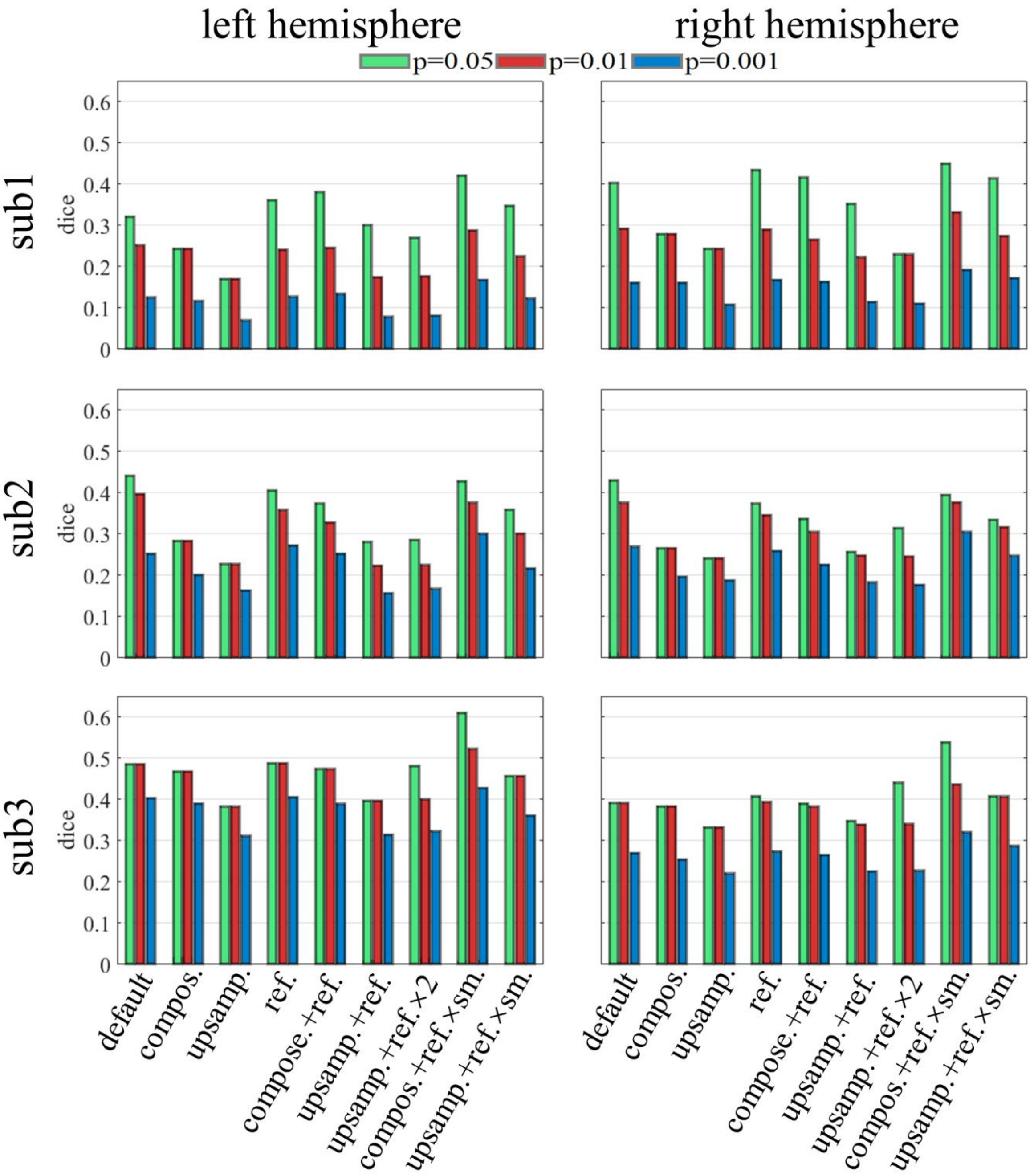

Fig. S3: Quantitative evaluation of overlap between "thin" and "thick" stripes using Dice coefficient for each subject. Each bar group shows overlap coefficient from different strategies. Each color represents a different statistical threshold used to binarize the activation maps. Less overlap between "thin" and "thick" stripes indicates less spatial blur. Abbreviations: "default" – Default pre-processing strategy with default surface vertex spacing and sequential spatial transformations. "compos" – Strategy of composing spatial transformations.

“upsamp” – Strategy of volume upsampling. “ref” – Strategy of surface mesh refinement (one iteration). “ref×2” – Strategy of surface mesh refinement (two iterations).
